# Autophagy impairment as a key feature for acetaminophen-induced ototoxicity

**DOI:** 10.1038/s41419-020-03328-6

**Published:** 2021-01-04

**Authors:** Tong Zhao, Tihua Zheng, Huining Yu, Bo Hua Hu, Bing Hu, Peng Ma, Ying Yang, Naidi Yang, Juan Hu, Tongtao Cao, Gang Chen, Bin Yan, Melina Peshoff, Maria Hatzoglou, Ruishuang Geng, Bo Li, Qing Yin Zheng

**Affiliations:** 1 Hearing and Speech Rehabilitation Institute, College of Special Education, Binzhou Medical University, Yantai, China; 2 Center for Hearing and Deafness, University at Buffalo, Buffalo, NY USA; 3 Department of Otolaryngology, Shenzhen Second People’s Hospital, The First Affiliated Hospital of Shenzhen University, Shenzhen, China; 4 Department of Genetics, School of Pharmacy, Binzhou Medical University, Yantai, China; 5 Key Laboratory of Flexible Electronics & Institute of Advanced Materials, Nanjing Tech University, Nanjing, China; 6 Department of Otolaryngology-Head & Neck Surgery, Second Affiliated Hospital, Xi’an Jiaotong University School of Medicine, Xi’an, China; 7 School of Pharmacy, Guangdong Pharmaceutical University, Guangzhou, China; 8 Department of Otolaryngology, Case Western Reserve University, Cleveland, OH USA; 9 Department of Genetics, Case Western Reserve University, Cleveland, OH USA

**Keywords:** Macroautophagy, Hair cell

## Abstract

Macroautophagy/autophagy is a highly conserved self-digestion pathway that plays an important role in cytoprotection under stress conditions. Autophagy is involved in hepatotoxicity induced by acetaminophen (APAP) in experimental animals and in humans. APAP also causes ototoxicity. However, the role of autophagy in APAP-induced auditory hair cell damage is unclear. In the present study, we investigated autophagy mechanisms during APAP-induced cell death in a mouse auditory cell line (HEI-OC1) and mouse cochlear explant culture. We found that the expression of LC3-II protein and autophagic structures was increased in APAP-treated HEI-OC1 cells; however, the degradation of SQSTM1/p62 protein, the yellow puncta of mRFP-GFP-LC3 fluorescence, and the activity of lysosomal enzymes decreased in APAP-treated HEI-OC1 cells. The degradation of p62 protein and the expression of lysosomal enzymes also decreased in APAP-treated mouse cochlear explants. These data indicate that APAP treatment compromises autophagic degradation and causes lysosomal dysfunction. We suggest that lysosomal dysfunction may be directly responsible for APAP-induced autophagy impairment. Treatment with antioxidant *N*-acetylcysteine (NAC) partially alleviated APAP-induced autophagy impairment and apoptotic cell death, suggesting the involvement of oxidative stress in APAP-induced autophagy impairment. Inhibition of autophagy by knocking down of *Atg5* and *Atg7* aggravated APAP-induced ER and oxidative stress and increased apoptotic cell death. This study provides a better understanding of the mechanism responsible for APAP ototoxicity, which is important for future exploration of treatment strategies for the prevention of hearing loss caused by ototoxic medications.

## Introduction

Acetaminophen (*N*-acetyl-p-aminophenol [APAP], also known as paracetamol) is widely used as a pain reliever and fever reducer. APAP is effective and safe at the therapeutic dosage. However, APAP overdose can cause severe hepatotoxicity in experimental animals and humans^1,2^. APAP-induced hepatotoxicity has been recognized for >50 years and its underlying mechanism is fairly well known. APAP is metabolized by a cytochrome P450 2E1 isozyme (CYP2E1) to a reactive intermediate, *N*-acetyl-p-benzoquinone imine (NAPQI). NAPQI causes the depletion of glutathione and the formation of APAP–cysteine adducts^3,4^. The APAP adducts lead to mitochondrial dysfunction and oxidative stress, which is a critical step in triggering hepatocyte necrosis and liver injury^5,6^. A previous study indicated that APAP adducts are removed through selective autophagy in primary mouse hepatocytes. The pharmacological induction of autophagy may be a novel and promising approach for treating APAP-induced liver injury^7^.

Autophagy, meaning “self-eating” in Greek, is an important cellular degradation process that occurs in response to physiological or chemical stresses^8,9^. Autophagy activation generates autophagosomes, which subsequently fuse with lysosomes to form autolysosomes. The lysosomal hydrolases degrade intra-autophagosomal contents to produce amino acids, which are used for the synthesis of macromolecules and for the production of energy^10^. Microtubule-associated protein 1A/1B light chain 3 (LC3) is ubiquitous in mammalian cells and tissues. LC3-II (a cleaved and phospholipid-conjugated form of LC3) and SQSTM1/p62 (sequestosome 1) are biomarkers for active autophagosomes. LC3-II is present in the membranes of autophagosomes and is degraded (along with p62, a cargo of autophagosomes) by lysosomal acidic enzymes after fusion of the lysosome and autophagosome. Incomplete maturation and a decrease in lysosome activity lead to decreased autolysosome formation and increased LC3-II and p62 accumulation in autophagosome membranes. Recent reports have shown that autophagy has protective effects, but impaired autophagy may induce cell death^7,11–14^.

Hair cells (HCs) in the cochlea play a critical role in converting mechanical signals into neural signals for hearing^15^ and are vulnerable to multiple types of damage, including acoustic trauma, ototoxicity, inflammation, and aging^16–20^. Autophagy plays an important role during the development of the inner ear and is essential for the survival of HCs under various ototoxic conditions^13,16,21–23^. In contrast to well-documented hepatotoxicity, APAP-induced ototoxicity has only recently been recognized in studies^24,25^. Clinical evidence indicates that the abuse of analgesics containing APAP/hydrocodone or APAP/codeine can cause hearing loss^26,27^. However, the mechanisms underlying the ototoxic effects of these drugs have yet to be fully characterized. Yorgason et al. reported that APAP, but not hydrocodone, was the primary ototoxic agent in neonatal mouse cochlear cultures and in a mouse HC-like auditory cell line (HEI-OC1), though the mechanism behind its toxic effects remains unknown^28^. Kalinec et al. investigated the mechanisms of APAP and NAPQI cytotoxicity in the HEI-OC1 cell line^29^. Their study suggested that APAP causes HEI-OC1 cell death by inducing oxidative stress, and the toxic effects of both APAP and NAPQI in HEI-OC1 cells involve the induction of endoplasmic reticulum (ER) stress signaling through controlling the protein kinase RNA-like ER kinases (PERK).

Furthermore, it has been suggested that an APAP-induced increase in autophagy could exert a cytoprotective effect against APAP-induced apoptosis in auditory cells^30^. Previous results have shown that Beclin-1 and LC3-II proteins increase following APAP exposure. However, the expression levels of the autophagy protein SQSTM1/p62, a cargo receptor involved in the selective degradation of ubiquitinated aggregates/organelles by autophagy, has not yet been determined in APAP-treated auditory cells. Thus, the autophagy mechanisms responsible for APAP ototoxicity in auditory cells remain unclear.

Therefore, we investigated whether the autophagy pathway plays a cytoprotective or cytotoxic role in APAP ototoxicity using two in vitro models: the HEI-OC1 cell line and mouse cochlear explants. We also examined the relationships between autophagy, oxidative stress, ER stress, and apoptosis signaling crosstalk in auditory cells. Our study offers novel insights into the APAP-induced ototoxicity mechanism and has the potential to lead to future treatment strategies for the prevention of APAP-induced hearing loss.

## Materials and methods

### Cell culture

The HEI-OC1 cell line was kindly provided by Professor Federico Kalinec (House Ear Institute, Los Angeles, CA, USA). Mouse auditory cell lines (House Ear Institute-Organ of Corti 1, HEI-OC1 cells) were cultured under permissive conditions (33 °C, 10% CO_2_) in high-glucose Dulbecco’s modified Eagle’s medium (DMEM; Gibco, #11965092) containing 10% fetal bovine serum (FBS; Gibco, #10437028) without antibiotics^30^.

### Cell Counting Kit-8 (CCK-8) cell viability assay

Cell viability was detected using a CCK-8 assay kit (Dojindo Laboratories, Kumamoto, Japan). Briefly, HEI-OC1 cells were trypsinized and collected. The cells were counted using an automated cell counter (Invitrogen Countess, Bothell, WA, USA), and the concentration was adjusted to 1.3 × 10^5^ cells/ml. The cells were then seeded into a 96-well cell culture plate (100 μl per well) and incubated overnight for attachment. The cells were then treated with APAP (MedChemExpress, HY-66005) in a dose-dependent manner (2, 4, 10, 25, 50 mM). After 24 h, a CCK-8 assay was performed following the manufacturer’s protocol. Subsequently, a microplate reader using the Gene5 software (BioTek Instruments, Winooski, VT, USA) measured the absorbance at 450 nm. The average optical density (OD) value in the control cells was used as the baseline of 100% viability. The half-maximal inhibitory concentration (IC50) was calculated using the GraphPad Prism 6 software.

### Drug administration

Chloroquine (CQ, Sigma-Aldrich, C6628) were used as the lysosomal inhibitor. The cell line was pretreated with CQ for 5 h, and APAP was added for the following experiments. Bafilomycin A1 (Baf, Sigma-Aldrich, B1793) was used to block the fusion of autophagosomes with lysosomes. The cells were cotreated with Baf (100 nM) and APAP for 24 h. Acetylcysteine (NAC, MedChemExpress, HY-B0215) was used to inhibit the reactive oxygen species (ROS) accumulation. 4-Phenylbutyric acid (4-PBA; Sigma-Aldrich, P21005) was used as an inhibitor of ER stress. After pretreated with 2 mM NAC or 1 mM 4-PBA for 4 h, the cells were exposed to APAP for 24 h.

### Crystal violet staining

Cell viability was also determined by crystal violet staining as previously described^31^. In brief, HEI-OC1 cells were seeded into a 24-well cell culture plate. After the designated treatment, the cells were washed in ice-cold phosphate-buffered saline (PBS; Meilunbio, MA0015) and fixed in cooled methanol on ice for 10 min. The cells were then stained with crystal violet solution (0.5% crystal violet in 25% methanol) for 10 min at room temperature. The cells were washed with tap water and allowed to dry. Stained cells were then extracted with 1% sodium dodecyl sulfate. After solubilization, the 100 μl per well dye extracts were measured at 570 nm using a microplate reader. The average OD value in the control cells was taken as 100% viability.

### Measurement of cell apoptosis by flow cytometry

HEI-OC1 cells were seeded in a 6-well cell culture plate at a density of 4 × 10^5^ cells/ml (2 ml per well) and incubated overnight. The cells were then treated with APAP at the concentration of either 10 or 20 mM or with the vehicle solution (0.5% dimethyl sulfoxide (DMSO), containing a volume of DMSO equal to that used in 20 mM APAP) for 24 h. Apoptosis was quantified using an Annexin V-FITC Apoptosis Detection Kit I (BD Biosciences, 556547) according to the manufacturer’s protocol. The stained cells were analyzed by a flow cytometer (FACSCanto II, BD Biosciences, San Jose, CA, USA).

### Mice

The study involved the use of the C57BL/6J (B6) inbred mouse strain. B6 mice at 6–8 weeks of age were obtained from the Model Animal Research Center (Nanjing, China). In addition, green fluorescent protein (GFP)-LC3 mice (#027139), obtained from the Jackson Laboratory, were used. Polymerase chain reaction (PCR) genotyping of the GFP-LC3 mice was performed according to the Jackson Laboratory’s recommendation. The primers used for genotyping are shown in Table 1 (in the supplemental file). The Animal Use and Care Committee of Binzhou Medical University approved all mouse experimental protocols. Animals were randomly distributed over the different experimental conditions and blinding was applied during data acquisition and analysis when possible.

### Cochlear explant culture

The cochlear explant culture procedures were performed as previously described^32,33^. In brief, mice at postnatal day 3 (P3) were euthanized after antisepsis with 70% ethanol, and the inner ears were removed. The cochleae were immersed in cold Hank’s balanced salt solution (HBSS; Solarbio, #H1045-500), and the organ of Corti and spiral ganglion neurons (SGNs) were carefully separated. The explants were placed onto a glass-bottomed dish coated with tissue adhesion solution (Corning, #354241), then washed by HBSS. Warmed culture medium (98% DMEM, 1% N-2, Thermo Fisher Scientific, #17502-048) and 1% ampicillin (Invitrogen, #11593-027) were added directly onto the explants. The cochlear explants were incubated (37 °C, 5% CO_2_) for 10–16 h, and then the final culture media (97% DMEM, 1% FBS, 1% N-2, and 1% ampicillin) was added to submerse the explants. APAP (5 or 10 mM) was added, and the tissue was incubated for 24 h to induce damage to the HCs of the explant-cultured cochleae.

### Mouse cochlear HC counts

After the explant cultures were treated with 5 or 10 mM APAP for 24 h, the HCs were stained with phalloidin-Alexa 488 (Invitrogen, A12379) to stain hair bundles and with 4,6-diamidino-2-phenylindole (DAPI; Invitrogen, D1306) to stain nuclei. The HCs were counted over a length of 150 μm from the apical to the basal turns of the cochlea. For each experimental condition, at least four organ of Corti samples were analyzed.

### Immunofluorescence staining

The cochlear explants were fixed for 30 min in 4% paraformaldehyde, washed 3 times for 5 min each with PBS, and blocked with 5% goat serum in PBS for 1 h. The tissues were then incubated with a primary antibody overnight at room temperature in blocking buffer. Then the tissues were washed 3 times for 5 min each with PBS and incubated with a secondary antibody in PBS for 1 h. The tissues were washed 3 times in PBS and counterstained with DAPI. Finally, the tissues were mounted on slides in ProLong^TM^ Gold Antifade Reagent (Cell Signaling Technology, #9071). The following antibodies were used in this study: anti-cleaved caspase 3 (Cell Signaling Technology, #9664, 1:400), anti-Myo7A antibody (Proteus BioSciences, #25-6790, 1:150), anti-p62 (Abcam, ab56416, 1:50), anti-LAMP1 (Abcam, ab24170, 1:200), and anti-p-S6 (Cell Signaling Technology, #4858, 1:100). The stained tissues were imaged using a confocal microscope (LSM 880, Zeiss, Germany). The clock scan protocol was used for image analysis by ImageJ (National Institutes of Health, Bethesda, MA, USA) as previously described^34^.

### Quantification of the GFP-LC3 immunofluorescence signals in cultured cochlear explant tissues

The GFP-LC3 fluorescent puncta were quantified as previously described^13^. After the indicated treatment of the cultured cochlear tissues, we counted the GFP-LC3-fluorescent puncta in each HC and the number of HCs along the entire cochlear length to obtain the average number of GFP-LC3-fluorescent puncta per HC for each cochlea. At least three independent cochleae were counted.

### Western blotting

HEI-OC1 cells were lysed with a RIPA lysis and extraction buffer (ThermoFisher Scientific, 89900) containing a protease and phosphatase inhibitor cocktail (Roche Diagnostics, Complete, 11697498001; PhosSTOP, 04906845001). The protein concentration was quantified using a BCA Protein Assay Kit (Takara, T9300A). Loading buffer (Takara, 9172) was added to the protein extract, and the sample was boiled for 5 min to denature the proteins. The protein extracts (30–50 μg) were then separated on polyacrylamide gels and transferred to polyvinylidene fluoride membranes (Merck Millipore, IPVH00010) for 40 min at 15 V using a semidry transfer system (Bio-Rad, Hercules, CA, USA). The membranes were blocked with 5% nonfat dried milk in Tris-buffered saline containing 0.1% Tween 20 (TBS-T) for 1 h at room temperature. The membranes were then probed overnight at 4 °C with relevant primary antibodies: anti-cleaved caspase-3 (Cell Signaling Technology, #9664), anti-Bcl-xL (Cell Signaling Technology, #2764), anti-BiP (Proteintech, 11587-1-AP), anti-CHOP (Proteintech, 15204-1-AP), anti-ATF4 (Proteintech, 10835-1-AP), anti-ATF6 (ABclonal, A0202), anti-XBP-1s (Cell Signaling Technology, #12782), anti-Caspase12 (Cell Signaling Technology, #2202), anti-HO-1 (Abcam, ab13248), anti-Nrf2 (Abcam, ab62352), anti-LC3B (Novus Biologicals, NB100-2220), anti-p62 (Abcam, ab56416), anti-p-AMPK (Cell Signaling Technology, #2531), anti-AMPK (Abcam, ab80039), anti-Hamartin/TSC1 (Cell Signaling Technology, #4906), anti-p-S6 (Ser235/236) (Cell Signaling Technology, #4858), anti-S6 (Cell Signaling Technology, #2217), anti-LAMP1 (Abcam, ab24170), anti-LAMP2 (Proteintech, 10397-1-AP), anti-Rab7 (Cell Signaling Technology, #2094), anti-TFEB (Cell Signaling Technology, #32361), anti-Atg7 (Cell Signaling Technology, #8558), anti-Atg5 (Cell Signaling Technology, # 12994), anti-Syntaxin17 (Proteintech, 17815-1-AP), anti-VAMP8 (Proteintech, 15546-1-AP), anti-SNAP29 (Proteintech, 12704-1-AP), and anti-β-actin (Proteintech, 20536-1-AP). After washing with TBS-T, the membranes were probed again for 1 h at room temperature with a species-specific secondary antibody (anti-rabbit, Abcam ab6728 or anti-mouse, Abcam ab6721) coupled to horseradish peroxidase. The immunoreactive bands were detected using a Chemiluminescent HRP Substrate Kit (Bio-Rad, #1705062, Clarity Max Western ECL Substrate) and visualized using the ChemiDoc XRS+ System (Bio-Rad). The intensities of the protein bands were measured and quantified using ImageJ, as described by Luke Miller (https://lukemiller.org/index.php/2010/11/analyzing-gels-and-western-blots-with-image-j/).

### MitoSOX staining

MitoSOX^TM^ Red (ThermoFisher, M36008) was used to analyze the mitochondrial ROS production. After the indicated treatment, the HEI-OC-1 cells were trypsinized and collected by centrifugation. The cells were resuspended in a solution containing 5 μM MitoSOX reagent, incubated for 10 min, washed with PBS, and analyzed by flow cytometry (FACSCanto II, BD Biosciences, San Jose, CA, USA). FlowJo V10 software was used to analyze the flow cytometric data. The explant-cultured tissues were also stained with 5 μM MitoSOX after the indicated treatment. The samples were imaged using a confocal microscope (LSM 880, Zeiss, Germany).

### LysoTracker staining

LysoTracker Red DND-99 (ThermoFisher, L7528) was used for the assessment of lysosome activity. This probe is highly selective for acidic organelles and is effective for labeling live cells^35^. After the indicated treatments, the HEI-OC1 cells were rinsed with PBS and stained with 100 nM LysoTracker Red in a serum-free medium for 30 min in an incubator at 37 °C. The cells were then washed with PBS. Lysosome size and staining intensity were viewed using a confocal microscope (LSM 880, Zeiss, Germany). Lysosome sizes were measured using the ImageJ software. At least 30 cells of a random area were measured in each group. After the indicated treatment, the explant-cultured tissues were also stained with 100 nM LysoTracker, and the samples were imaged using a confocal microscope (LSM 880, Zeiss, Germany).

### Magic Red staining

Cathepsin B activity was measured using the Magic Red Cathepsin B Detection Kit (ImmunoChemistry Technologies, 937) as previously described^12^. Briefly, control or APAP-treated HEI-OC1 cells were cultured in confocal dishes (for microscopy) for different lengths of times, as indicated. The cells were then loaded with Magic Red Cathepsin B reagent for 1 h, followed by washing twice with PBS. More than ten fluorescence images were taken using a confocal microscope and representative images are shown. ImageJ software was used to quantify the cathepsin B activity.

### Acridine orange staining

Lysosomal V-ATPase activity was assessed with acridine orange staining. Acridine orange uptake represents lysosomal V-ATPase-driven pumping of hydrogen ions into the lysosomes^36^. After the indicated treatments, HEI-OC1 cells were washed with PBS and incubated with 1 μM acridine orange (ThermoFisher, A1301) in the cultured medium for 30 min at 33 °C. The acridine orange was then removed, and the cells were examined under a confocal microscope (LSM 880, Zeiss, Germany). The cytoplasm and nuclei of stained cells showed bright green fluorescence, whereas the acidic autophagic vacuoles showed bright red fluorescence^10,37^.

### Plasmid transfection

Autophagy flux was monitored by fluorescence microscopy of the distribution and alteration of mRFP-GFP-LC3B fluorescence signals^38^. HEI-OC1 cells were seeded into glass-bottom dishes and transfected for 48 h with mRFP-GFP-LC3B plasmid (a gift from Dr. Yingyu Chen, Peking University, Beijing, China) using Lipofectamine 3000 transfection reagent (ThermoFisher Scientific, L3000015). After the designated treatments, the cells were fixed with 4% paraformaldehyde and examined under a confocal microscope. To quantify autophagic cells, GFP-LC3 and mRFP-LC3 punctate dots were determined from triplicate samples by counting >30 cells.

### Transmission electron microscopy

HEI-OC1 cells were fixed in 3% glutaraldehyde at 4 °C for 24 h, postfixed in 1% osmium tetroxide for 1.5 h, and dehydrated through a series of graded ethanol solutions. Fixed samples were embedded in Spurr’s resin (Electron Microscopy Sciences, 14300) and thin sections (80 nm) were cut. The sections were stained with uranyl acetate and lead citrate and observed under a JEM-1400 transmission electron microscope (JEOL, Tokyo, Japan) at the Medicine Research Center, Binzhou Medical University, Yantai, China. The numbers of lysosomes and autophagic vacuoles were quantified in each sample for at least ten cells and confirmed by two additional repetitions of the experiments.

### Transfection with small interfering RNA (siRNA)

The siRNA targeting mouse *Atg5* or *Atg7* and scrambled control siRNA were obtained from GenePharma (Shanghai). HEI-OC1 cells were transfected with 50 nM siRNA or negative control siRNA using Lipofectamine 3000 Transfection Reagent (Invitrogen) according to the manufacturer’s instructions. Seventy-two hours following transfection, the cells were exposed to 20 mM APAP for 24 h. The cells were analyzed by real-time cell analyzer (RTCA) or collected and processed for immunoblotting.

### Real-time cell analyzer

Cytotoxicity was monitored by the xCELLigence RTCA DP system (ACEA Biosciences, USA) as previously described^39^. First, the background of the E-plates was determined in 50 μl of medium, and 100 μl of the HEI-OC1 cell suspension was added (1.3 × 10^4^ cells per well). Cells were incubated for 30 min at room temperature, and E-plates were placed into the RTCA station. Cells were grown for at least 24 h, with impedance being measured every 15 min. After the designated treatments, cells were monitored again every 15 min until the end of the experiment. The electronic readout, cell-sensor impedance induced by adherent cells to the electron flow, is displayed as an arbitrary unit, known as the cell index. The normalized cell index was calculated by the RTCA software at the selected normalization time point, which was chosen as the time immediately before the addition of treated drugs. Each treatment was performed in triplicate.

### Statistical analysis

Each experiment was repeated at least three times. No samples or animals were excluded from the analysis. All data are presented as the mean ± SEM. Microsoft Excel and GraphPad Prism 6 software were used for data analysis. Unpaired Student’s *t* test was used to determine statistical significance when comparing two groups, and one-way analysis of variance (ANOVA) was used when comparing more than two groups. A value of *P* < 0.05 was considered statistically significant.

## Results

### APAP induces apoptotic cell death in HEI-OC1 cells and cochlear HCs

**Fig. 1:**
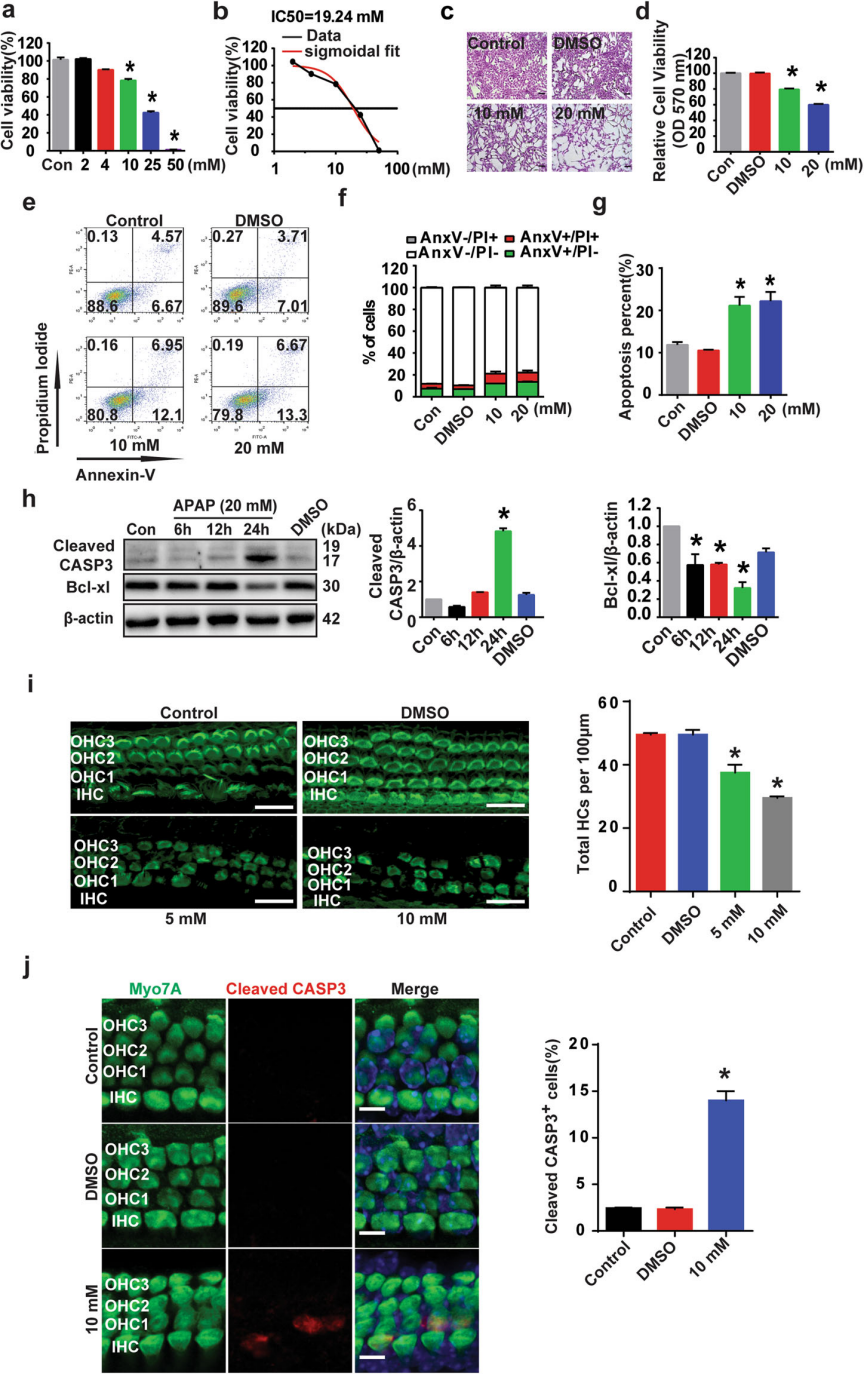
APAP induced apoptotic cell death in HEI-OC1 cells and cochlear HCs. **a** HEI-OC1 cells were treated with APAP (2, 4, 10, 25, 50 mM) for 24 h. The cell viability was detected by CCK-8 assay. *P < 0.05 vs. control cells (Con). **b** The IC50 of APAP treatment was 19.24 mM at 24 h. IC50 with the curve fit line and the IC50 value are illustrated. **c** The cell viability was also determined by the crystal violet assay. HEI-OC1 cells were treated with APAP (10, 20 mM) or vehicle (1% DMSO with the volume equal to that used in 20 mM APAP) for 24 h. The cells were then stained with crystal violet, and morphological alterations were observed under a light microscope. Scale bar = 0.86 mm. **d** The quantitative bar graph shows the relative density of cells from the crystal violet assay. *P < 0.05 vs. control cells. **e** HEI-OC1 cells were treated with 1% DMSO or APAP (10, 20 mM). The percentage of apoptotic cells was determined by flow cytometry using annexin V and PI labeling. The flow cytometric plots are representative of four experiments; mean ± SEM are shown in the bar plots (**f**), AnxV^+^/PI^−^ (green bar; early apoptotic; lower right quadrant), AnxV^+^/PI^+^ (red bar; late apoptotic; upper right quadrants), AnxV^−^/PI^−^ (white bar; live cells; lower left quadrant); and AnxV^−^/PI^+^ (gray bar; dead cells; upper left quadrants) are shown. **g** Quantification of the proportion of apoptotic cells (early and late apoptotic cells) after APAP treatment from the flow cytometric data. *P < 0.05 vs. control cells. **h** HEI-OC1 cells were treated with 20 mM APAP for a designated period (6, 12, or 24 h) or vehicle (1% DMSO) for 24 h. The protein expression of cleaved caspase-3 and Bcl-xl was detected by western blotting. The right two panels show the results of densitometric analysis. *P < 0.05 vs. control cells. **i** The cochlear explants treated with vehicle (0.5% DMSO) and 5 mM or 10 mM APAP for 24 h were stained for F-actin with phalloidin (green fluorescence). The images are representatives of four individual preparations. Confocal images were taken from the middle turn of the cochlea. Scale bar = 10 μm. OHC1, OHC2, and OHC3 represent the first, second, and third row of outer HCs, respectively; IHC inner HCs. The mean percentages of OHC loss are shown on the bar graph. Data are presented as the mean ± SEM (*n* = 3 mice per group). *P < 0.05 vs. control group. **j** The cochlear explants treated with vehicle (0.5% DMSO) or 10 mM APAP for 24 h. Apoptotic HCs were detected with a caspase 3 (CASP3) staining assay. Apoptotic cells were seen as caspase3-positive cells in the middle turns of cochlear explants from the APAP-treated groups. Scale bar = 5 μm. The right panel shows the comparison of the numbers of positive cells between the treated and control groups. *P < 0.05 vs. control group.

APAP-induced ototoxicity has been linked to cell death^29^. In this study, we examined the underlying mechanisms for APAP-induced auditory cell death. First, HEI-OC1 cells were exposed to APAP, and the cell viability was examined using CCK-8 and crystal violet staining assays. The CCK-8 assay revealed that APAP exposure decreased the cell viability in a dose-dependent manner at 24 h after treatment (Fig. 1a). Because the calculated IC50 of APAP was 19.24 mM (Fig. 1b), we used 20 mM APAP as the treatment concentration for the subsequent HEI-OC1 cell experiments. Crystal violet staining revealed significant cell shrinkage and decreased cell numbers after APAP treatment (Fig. 1c, d). Flow cytometry showed that APAP induced HEI-OC1 cell death via apoptosis (Fig. 1e). The proportions of apoptotic cells at both the early (AnxV^+^/PI^−^) and the late phase (AnxV^+^/PI^+^) after APAP treatment were significantly increased compared with the control cells (Fig. 1f, g). We also examined the expression of apoptosis-associated proteins (Fig. 1h). Western blotting showed that the protein expression of cleaved caspase 3 (CASP3) in HEI-OC1 cells was significantly increased and the protein expression of Bcl-xl was significantly decreased after 24 h of 20 mM APAP treatment compared with the control cells.

Next, we dissected the cochleae from postnatal day 3 (P3) mice and cultured the organ of Corti with 5 or 10 mM APAP for 24 h. The mice cochlear explants in the absence of APAP maintained their normal morphology at 24 h (Fig. 1i). In contrast, outer HC loss began from the middle segment of the organ of Corti after 24 h of incubation with 5 mM APAP and worsened with the increase in the concentration to 10 mM (close to the IC50). The morphology of the stereociliary bundles of outer HCs was significantly changed after APAP treatment. The cochlear explants may have been more sensitive to the APAP treatment than the HEI-OC1 cells. Therefore, we used 10 mM APAP as the treatment concentration of the subsequent mice cochlear explant experiments. Immunolabeling of cleaved CASP3, an apoptotic marker, also showed a significant increase in the number of CASP3-positive HCs in the APAP-treated cochlear explants compared with the control group (Fig. 1j). These results demonstrated that APAP induces apoptotic cell death in HEI-OC1 cells and cochlear HCs.

### APAP induces oxidative stress in HEI-OC1 cells and cochlear HCs

**Fig. 2:**
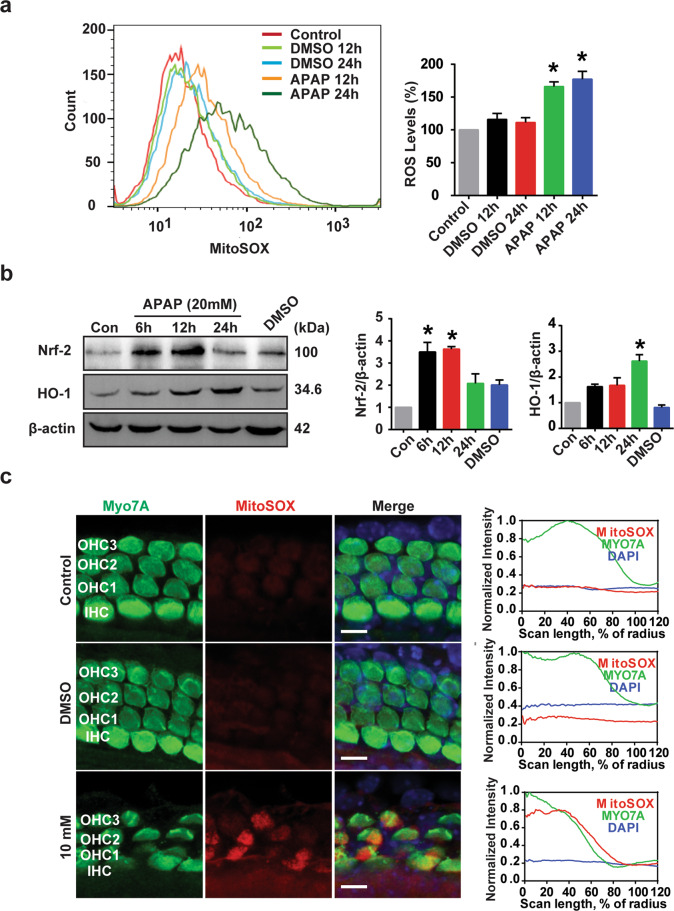
APAP induces oxidative stress in HEI-OC1 cells and cochlear HCs. **a** HEI-OC1 cells were treated with 20 mM APAP or vehicle (1% DMSO) for 12 or 24 h. HEI-OC1 cells were then analyzed for mitochondrial superoxide generation by flow cytometry using MitoSOX Red. Representative line graphs are shown in the left panel. Quantification plots are shown in the right panel. The results were calculated as a percentage of normal control fluorescence intensity. Bars are the mean ± SEM (*n* = 3). *P < 0.05 vs. control cells. **b** HEI-OC1 cells were treated with 20 mM APAP for the indicated periods (6, 12, or 24 h) or vehicle (1% DMSO) for 24 h. The protein expression of Nrf-2, HO-1, and β-actin was measured by western blotting. The right panel shows the results of the densitometric analysis. *P < 0.05 vs. control cells. **c** Immunofluorescence staining with Mito-SOX in the middle turn of the cochlea after treatment with 0.5% DMSO or 10 mM APAP for 24 h. Scale bar = 5 μm. Fluorescence intensities were quantified using the clock scan protocol in ImageJ.

The cytotoxic effects of APAP might be associated with oxidative stress in HEI-OC1 cells because APAP significantly increased the production of ROS in these cells^29^. We used a mitochondrial superoxide indicator, MitoSOX, to detect the mitochondrial ROS level in APAP-treated HEI-OC1 cells. The mitochondrial ROS levels significantly increased after APAP treatment compared with those of control groups at 12 and 24 h (Fig. 2a). Because ROS act as key mediators in APAP ototoxicity models, the antioxidant defense mechanism may be necessary to maintain normal cellular function. Thus, we examined the expression of oxidative stress-related proteins. Western blotting analysis revealed that Nrf-2 (NF-E2-related factor-2) and its dependent protein, HO-1 (heme oxygenase-1), both had higher expression levels when cells were exposed to APAP compared with control cells at 6 and 12 h (Fig. 2b). The Nrf-2 protein expression returned to the baseline at 24 h after APAP treatment, but HO-1 protein expression remained at a higher expression level than in control cells at 24 h after APAP treatment. The results suggest that the activation of the Nrf-2/HO-1 signaling pathway may contribute to the APAP-induced oxidative stress. MitoSOX staining in the cochlear HCs also showed that the ROS level was significantly increased by APAP treatment (Fig. 2c). The results demonstrated that APAP induces oxidative stress in HEI-OC1 cells and in cochlear HCs.

### APAP induces ER stress in HEI-OC1 cells

It has been reported that the cytotoxic effects of APAP on HEI-OC1 cells involve the activation of ER stress signaling by controlling the PERK-mediated branch of this signaling pathway^29^. Here we found that another branch of the ER stress response was also involved in APAP ototoxicity. We investigated the expression of several ER stress-inducible UPR proteins (Fig. S1a). APAP induced time-dependent increases in the protein expression levels of BiP (immunoglobulin-binding protein) and CHOP (CCAAT/enhancer-binding protein homologous protein) in HEI-OC1 cells. APAP treatment also increased the expression of ATF-4 protein at the early time point (6 h), suggesting that APAP affects UPR events initiated by the stress sensor, PERK. To define the possible regulation of another stress sensor (IRE1α) by APAP, we examined the levels of IRE1α’s downstream target X-box-binding protein-1 (XBP-1) in HEI-OC1 cells through western blotting and reverse transcription (RT)-PCR. The protein expression of spliced isoform XBP-1 (XBP-1s) was transiently enhanced at 6 h after APAP treatment but was subsequently decreased after 12 h of APAP treatment. Consistent with this finding, RT-PCR analysis revealed a significant increase in the levels of XBP-1 mRNA splicing in HEI-OC1 cells in the early time points after APAP treatment (Fig. S1b), indicating that APAP enhances the IRE1α activity. Together, these results suggest that APAP induces the ER stress response in HEI-OC1 cells through PERK and IRE1α, two UPR branches.

### APAP impairs degradation of autophagosome cargo in HEI-OC1 cells and cochlear HCs

**Fig. 3:**
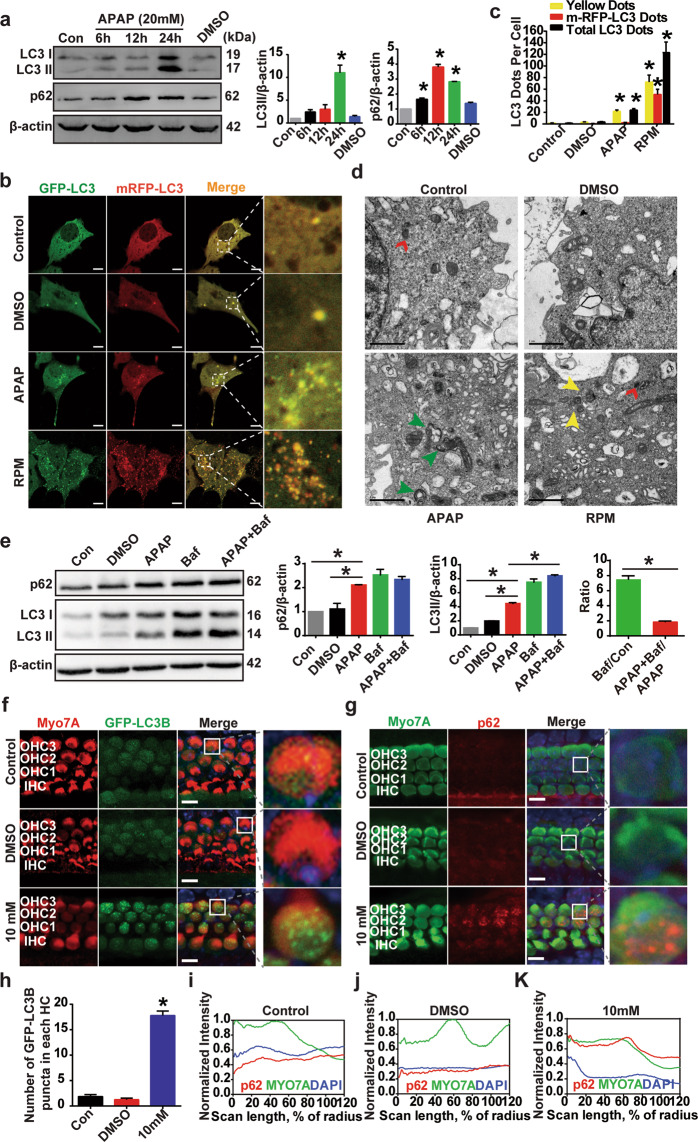
APAP impairs degradation of autophagosome cargo in HEI-OC1 cells and cochlear HCs. **a** HEI-OC1 cells were treated with 20 mM APAP for the indicated periods (6, 12, 24 h) or vehicle (1% DMSO) for 24 h. The protein expression of LC3, p62, and β-actin was measured by western blotting. The right two panels show the results of the densitometric analysis *P < 0.05 vs. control cells. **b** HEI-OC1 cells were transiently transfected with mRFP-GFP-LC3 plasmids for 48 h, and then the cells were treated with vehicle (1% DMSO), 20 mM APAP, or 0.4 μM rapamycin (RPM) for 24 h. Red and green puncta were visualized by confocal microscopy. Enlarged images outlined by dashed lines illustrate punctate fluorescence. Scale bar = 10 μm. **c** The bar graph shows the quantification of the ratio of mRFP-GFP-LC3 red to yellow puncta. *P < 0.05 vs. control cells. **d** Lysosome and autophagic vacuoles (AVs) were analyzed by transmission electron microscopy in HEI-OC1 cells treated with DMSO, APAP, or RPM. Representative transmission electron micrographs of cell-in-cell structures showing autophagosomes (green arrowheads), lysosomes (red arrowheads), and autolysosomes (yellow arrowheads). Scale bar = 1 μm. **e** Western blot with anti-p62 and anti-LC3 antibodies after APAP and Baf treatment. *P < 0.05. **f** Immunofluorescence staining with Myo7A antibody in cochlear explants from GFP-LC3 mice. Representative images are shown. Confocal images were taken from the middle turn. Enlarged images outlined by dashed lines illustrate punctate fluorescence. Scale bar = 5 μm. **g** Cochlear explants treated with vehicle (0.5% DMSO) or 10 mM APAP for 24 h were immunostained by Myo7A and p62 antibodies. The images are representative of four individual preparations. Confocal images were taken from the middle turn. Enlarged images outlined by white lines indicate p62 fluorescence in outer HCs. Scale bar = 5 μm. Fluorescence intensities were quantified using the clock scan protocol with ImageJ. The line plots are shown in **i**–**k**. **h** Quantification of the GFP-LC3 punctum number from **f**. *P < 0.05 vs. control cells. OHC1, OHC2, and OHC3 represent the first, second, and third row of outer HCs, respectively; IHC inner HCs.

In addition to induction of oxidative stress and ER stress, APAP treatment affects autophagy^30^. However, the role of autophagy in APAP-induced ototoxicity is not yet clear. In this study, we investigated the effect of APAP on this cellular degradation phenomenon. First, we determined the protein expression of an autophagosome marker, LC3, and an autolysosome marker, p62, in HEI-OC1 cells with or without APAP treatment. A time-dependent increase in LC3-II expression was observed in APAP-treated cells compared to control cells (Fig. 3a). LC3-II accumulation was increased at 24 h after APAP treatment. The p62 protein expression was also increased in a time-dependent manner after APAP treatment. To confirm autophagosome and autolysosome formation, we transfected HEI-OC1 cells with the GFP-LC3 plasmid and tandem mRFP-GFP-LC3 plasmid as reporter of autophagic flux^40^. We found that the numbers of GFP-LC3 puncta were significantly increased in HEI-OC1 cells after 24 h APAP exposure (Fig. S2). Autophagosomes that have not fused with lysosomes appear as yellow (mRFP and GFP) puncta, whereas autolysosomes appear as red (mRFP) puncta. The number of autolysosomes was markedly increased in the rapamycin-treated cells (positive autophagic control, rapamycin is a mammalian target of rapamycin (mTOR)-dependent autophagy activator) compared with control cells (Fig. 3b, c). The number of autophagosomes, but not autolysosomes, increased in APAP-treated cells compared with control cells. To further confirm the stage of inhibition on autophagy, we conducted autophagy flux assay. The blockage of autophagy flux with Baf showed that autophagosome synthesis is not increased with APAP treatment, because there is no difference in LC3-II protein level between Baf treatment and Baf plus APAP. On the other hand, it seems that the autophagy flux is reduced with APAP since the ratio of LC3-II APAP+Baf to LC3-II APAP is smaller than that of LC3-II Baf to LC3-II control, which explains the accumulation of LC3-II and p62 in the presence of APAP (Fig. 3e). Transmission electron microscopy was used to visualize the ultrastructures of autophagy organelles in HEI-OC1 cells (Fig. 3d). Taken together, the data demonstrate that APAP decreases the autolysosome formation in HEI-OC1 cells. Furthermore, these results indicate that APAP impairs the autophagy process, which might be associated with ER stress, oxidative stress, and cell death mechanism.

We also examined the autophagy process in mice cochlear explants after treatment with 10 mM APAP for 24 h. GFP-LC3 mice were used to examine the autophagosomes accumulation after APAP treatment. The cochleae were dissected from P3 GFP-LC3 mice and immunolabeled with an antibody for the HC marker myosin VIIA (Myo7A) after culturing the cochleae with 10 mM APAP for 24 h. The numbers of LC3 puncta were significantly higher in HCs after 24 h of APAP treatment compared with the controls (Fig. 3f, h). The cochlear explants were immunolabeled with an anti-p62 antibody after culturing the cochleae with 10 mM APAP for 24 h. The p62 protein expression was increased after APAP treatment (Fig. 3g, i–k). These results indicate that APAP induces autophagy impairment in mice cochlear explants. To investigate how the autophagy degradation process was impaired or blocked after APAP treatment, we assessed the protein expression of Rab7, Syntaxin 17, SNAP29, and VAMP8, which are necessary for the fusion of autophagosomes with lysosomes^41^. A reduction of SNAP29 levels but no detectable alteration in Rab7, Syntaxin 17, and VAMP8 levels was found in HEI-OC1 cells after treatment with APAP (Fig. S3). These results suggested that APAP may impair the fusion of autophagosomes with lysosomes by decreasing SNAP29.

### APAP-induced transient AMPK activation and mTORC1 suppression in HEI-OC1 cells and cochlear HCs

**Fig. 4:**
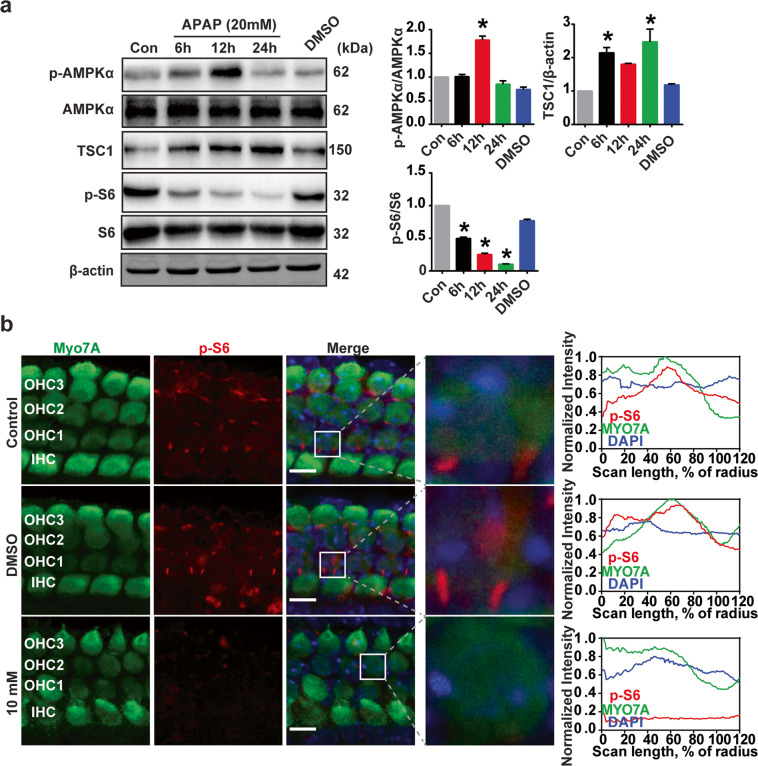
APAP induces AMPK activation and mTORC1 suppression in HEI-OC1 cells and cochlear HCs. **a** The protein expression of p-AMPKα, AMPKα, TSC1, p-s6, s6, and β-actin was measured by western blotting. The three panels on the right show the results of densitometric analysis. *P < 0.05 vs. control cells. **b** Cochlear explants treated with vehicle (0.5% DMSO) or 10 mM APAP for 24 h were immunostained by Myo7A and p-s6 antibodies. The images are representative of four individual preparations. Confocal images were taken from the middle turn. Enlarged images outlined by white line indicate p-s6 fluorescence in outer HCs. Scale bar = 5 μm. Fluorescence intensities were quantified using the clock scan protocol in ImageJ.

The roles of AMPK and mTORC1 in autophagy could depend on cell types and metabolic conditions^42^. To find out whether AMPK and mTORC1 signaling are involved in APAP-induced autophagy impairment, the phosphorylation of AMPK and mTORC1 substrate, S6 ribosomal protein (S6) phosphorylation at 235/236 (p-S6), was examined. APAP treatment resulted in a significant time-dependent decrease in the levels of p-S6 compared with control cells (Fig. 4a). Immunoreactivity of p-S6 in the middle-turn outer HCs decreased in the APAP group, suggesting an inhibition of mTORC1 activity (Fig. 4b). AMPK activity was increased transiently as shown by increased phospho-AMPK level at 6 and 12 h after APAP treatment but decreased later (Fig. 4a). However, the inhibition of mTORC1 and increased activity of AMPK did not cause significant change in autophagosome formation as shown in autophagy flux assay. The meaning of APAP-induced changes of AMPK and mTORC1 signaling needs to be further studied by examining the phosphorylation and activities of other downstream factors, especially the ULK1 complex (ULK1-PB1CC1/FIP200-ATG13-ATG101).

### APAP induces lysosome dysfunction in HEI-OC1 cells

**Fig. 5:**
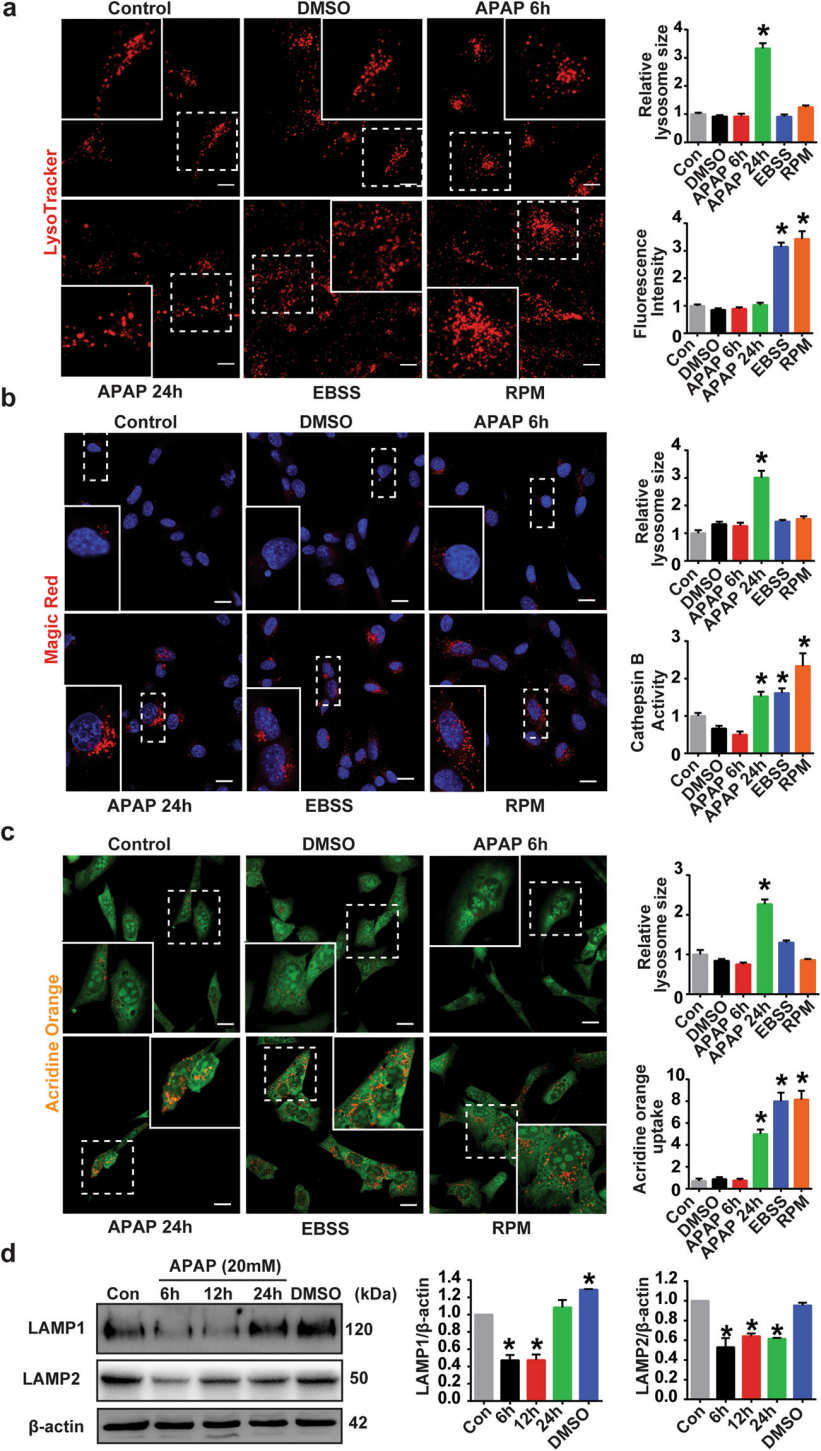
APAP inhibits lysosome function in HEI-OC1 cells. **a**–**c** HEI-OC1 cells were treated with vehicle (1% DMSO) or 20 mM APAP for 6 or 24 h. The positive control cells were treated with EBSS for 2.5 h or with 0.4 μM rapamycin (RPM) for 24 h. The cells were exposed to 100 nM LysoTracker or Magic Red reagent (**b**) or 1 mg/ml acridine orange (**c**). Images were taken by confocal microscopy. Representative images and enlarged images outlined by white lines are shown; scale bar = 5 μm. Relative lysosome sizes and fluorescence intensities were quantified using ImageJ. *P < 0.05 vs. control cells. **d** HEI-OC1 cells were treated with vehicle (1% DMSO) or 20 mM APAP for the indicated periods (6, 12, 24 h). Protein expression of LAMP1, LAMP2, and β-actin was measured by western blotting. The right panel shows the results of the densitometric analysis. *P < 0.05 vs. control cells.

Lysosomal activity is important for the autophagy degradation process^12,43^. To investigate the APAP-induced autophagy impairment mechanism, we examined the function of lysosomes using a few lysosome indicators and the protein expression levels of key lysosome enzymes. First, using a lysosomal dye (LysoTracker Red) we found that the lysosomal fluorescence intensity did not increase in the APAP-treated HEI-OC1 cells compared with the control cells at 6 and 24 h (Fig. 5a). To verify the validity of our assay, we repeated the analysis in positive control samples. The lysosomal fluorescence intensity increased in the positive control cells (EBSS and rapamycin-treated cells). A lysosomal hydrolase, cathepsin B activity, was then measured using Magic Red reagent. We found that APAP-treated cells at the early treatment time did not have increased cathepsin B activity, but they showed increased activity at 24 h after APAP treatment (Fig. 5b). Consistent with these findings, staining with acridine orange, a lysosomal V-ATPase activity indicator, showed that APAP-treated cells did not have increased lysosomal V-ATPase activity at the early treatment time and that the activity increased only after a prolonged treatment (24 h) (Fig. 5c). The 3 staining methods revealed that the relative lysosome size increased significantly in the APAP-treated cells at 24 h. These results suggest that treatment with APAP increases the number of enlarged acidic vesicles. We also measured the expression levels of lysosomal-associated membrane protein 1 (LAMP1) and LAMP2 (Fig. 5d). Both expression levels decreased at the early time point after APAP treatment and then slightly recovered with prolonged treatment. These findings suggested that the APAP-treated cells exhibited low lysosomal activity at the early time point, which was probably linked to the decreased autophagy-substrate clearance observed following APAP treatment.

We also examined transcription factor EB (TFEB), a master regulator of lysosomal biogenesis and autophagy^44^. The protein expression levels of TFEB significantly increased at the early treatment time but gradually returned to the baseline after a longer treatment time (Fig. S4a). HEI-OC1 cells transfected with TFEB plasmid showed stronger nuclear translocation of TFEB in the APAP-treated cells than in the control cells (Fig. S4b), suggesting that APAP induces lysosome biogenesis through TFEB activation. Moreover, we found enlarged vesicles accumulated around nuclei at the early time point after APAP treatment. This finding is consistent with the lysosome staining results in Fig. 5a–c in which the relative lysosome size was increased after APAP treatment.

### APAP induces lysosome dysfunction in cochlear HCs

**Fig. 6:**
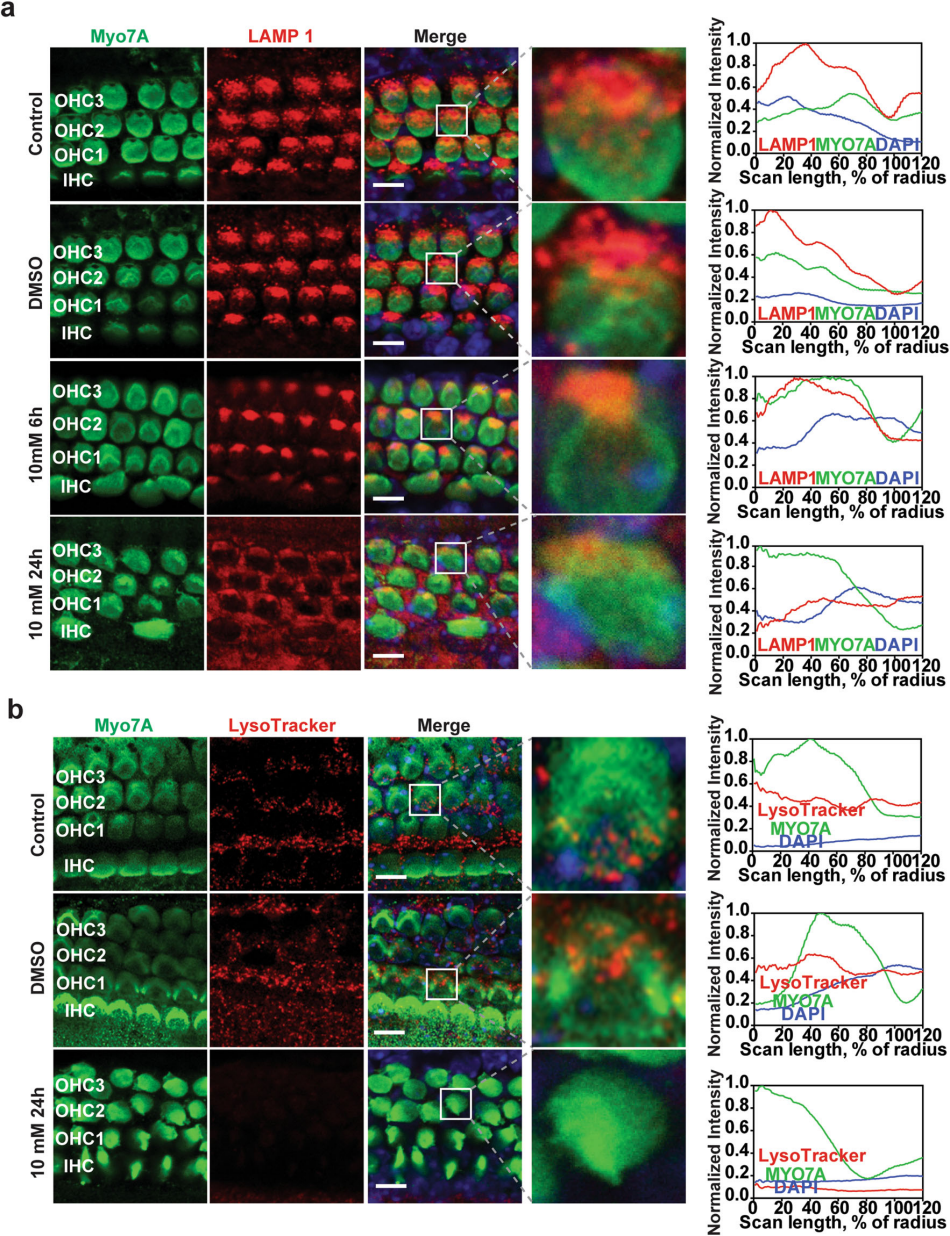
APAP inhibits lysosome function in cochlear HCs. **a** The cochlear explants treated with vehicle (0.5% DMSO) or 10 mM APAP for 6 or 24 h were immunostained for Myo7A (green fluorescence) and LAMP1 (red fluorescence). Enlarged images outlined by white lines indicate LAMP1 fluorescence in outer HCs. The images are representative of four individual preparations. Confocal images were taken from the middle turn. Scale bar = 5 μm. Fluorescence intensities were quantified using the clock scan protocol in ImageJ. **b** The cochlear explants treated with vehicle (0.5% DMSO) or 10 mM APAP for 24 h were stained by LysoTracker Red. Enlarged images outlined by white lines indicate LysoTracker fluorescence in outer HCs. The images are representative of four individual preparations. Confocal images were taken from the middle turn. Scale bar = 5 μm. Fluorescence intensities were quantified using the clock scan protocol by ImageJ. OHC1, OHC2, and OHC3 represent the first, second, and third row of outer HCs, respectively; IHC inner HCs.

The cochlear explants were immunolabeled with an anti-LAMP1 antibody after culturing the cochleae with 10 mM APAP for 6 or 24 h. LAMP1 protein expression decreased after APAP treatment in a time-dependent manner (Fig. 6a). Twenty-four hours after APAP treatment, the cytosolic LAMP1 diffused and distributed throughout the gap among HCs. The lysosomal fluorescence intensity (LysoTracker) decreased after APAP treatment (Fig. 6b). These results indicate that APAP may induce lysosomal impairment in mice cochlear explants.

### ROS signaling is involved in APAP-induced autophagy impairment

**Fig. 7:**
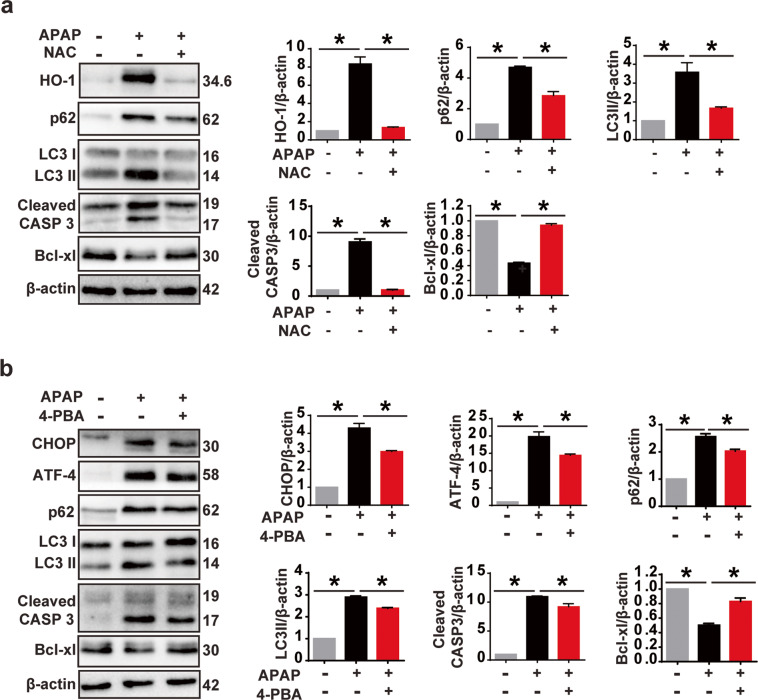
Inhibition of ER stress or oxidative stress in HEI-OC1 cells affect APAP-induced autophagy process and cell viability. **a** Western blots with anti-HO-1, anti-p62, anti-LC3, anti-cleaved CASP3, and anti-Bcl-xl antibodies after APAP and antioxidant treatment (NAC). The four panels on the right show the results of densitometric analysis. *P < 0.05. **b** Immunoblot analysis of APAP-induced change in the CHOP, ATF-4, p62, LC3, cleaved CASP3, and Bcl-xl in the presence of the ER stress inhibitor 4-PBA. The graphs show quantification of band intensities. *P < 0.05.

Several lines of evidence indicate that ROS and ER stress are the upstream modulators of autophagy and signal to the autophagic machinery in the presence of stimuli^45–48^. To investigate whether ROS is involved in APAP-induced autophagy impairment, we treated the HEI-OC1 cells with antioxidant NAC. Western blot results showed that, compared with the APAP-only group, the expression level of HO-1 was significantly decreased in NAC+APAP group, which suggested that APAP-induced ROS level was decreased in the pretreatment with NAC (Fig. 7a). Meanwhile, we observed a reduction in the levels of p62 and LC3-II in NAC-treated cells compared with the APAP-only group, indicating that NAC treatment attenuated APAP-induced autophagy impairment (Fig. 7a). Taken together, our results indicated the involvement of oxidative stress in APAP-induced autophagy impairment as well as apoptotic cell death. It is well known that ROS induces ER stress. We also observed a reduction of ER stress by NAC treatment (Fig. S5).

To understand the relationship between ER stress and autophagy in HEI-OC1 cells, we tested the effect of ER stress inhibitor 4-PBA on APAP-induced autophagy impairment and apoptotic cell death. Western blot results show a decrease in CHOP and ATF-4 in the 4-PBA+APAP group compared with APAP treatment alone, indicating that 4-PBA treatment partially suppressed APAP-induced ER stress (Fig. 7b). LC3-II and p62 protein levels were also slightly decreased in 4-PBA+APAP treatment compared to APAP alone, indicating 4-PBA has weak effect on the alleviation of APAP-induced autophagy impairment (Fig. 7b). Immunoblot analysis of Bcl-xl and cleaved CASP3 showed that 4-PBA treatment slightly attenuated APAP-induced apoptotic cell death (Fig. 7b). Weak effect was also seen in apoptotic signaling proteins but no effect was seen on cell survival (data not shown). This result suggested that ER stress has a weak, if any, contribution to APAP-induced autophagy impairment, and intervention on ER stress signaling was not an effective way to attenuate APAP-induced cell death.

### Inhibition of autophagy enhances APAP-induced ER stress, oxidative stress, and apoptotic cell death

**Fig. 8:**
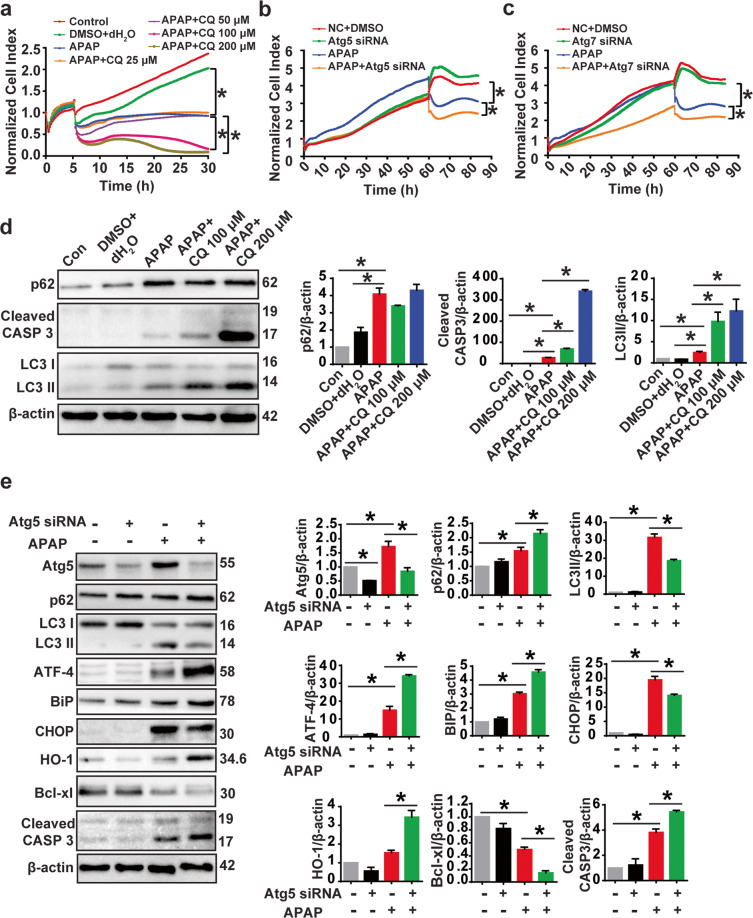
Chloroquine and *Atg5* deficiency in HEI-OC1 cells affect APAP-induced ER stress, oxidative stress, and cell viability. **a** RTCA showed that CQ aggravates APAP-induced apoptotic cell death. HEI-OC1 cells were treated with 100 μM and 200 μM CQ for 5 h before APAP treatment. *P < 0.05. **b**, **c** Knockdown of *Atg5* and *Atg7* aggravates APAP-induced apoptotic cell death tested by RTCA. *P < 0.05. **d** Western blot results showed that the lysosomal inhibitors CQ and APAP co-treatment inhibits autophagic flux and induces cell apoptosis in HEI-OC1 cells compared to APAP alone. The three panels on the right show the results of densitometric analysis. *P < 0.05. **e** Western blot analysis of oxidative stress, ER stress, autophagy, and apoptosis-related protein expression in the *Atg5* knockdown group after APAP injury. *P < 0.05.

To determine the role of autophagy in APAP-induced oxidative stress, ER stress, and cell death in HEI-OC1 cells, we tested the effects of knocking down autophagy genes (*Atg5* and *Atg7*) and lysosomal inhibitor that disrupts the fusion of autophagosome and lysosome, in the presence of APAP, on ER stress, oxidative stress, and apoptotic cell death in HEI-OC1 cells. RTCA and immunoblot analysis of cleaved CASP3 showed that CQ+APAP aggravates apoptotic cell death compared to APAP alone group (Fig. 8a, d). CQ+APAP significantly increased LC3-II protein level compared with APAP alone group in HEI-OC1 cells (Fig. 8d). However, there was no significant change in the accumulation of p62 in the presence of CQ compared to the APAP alone group (Fig. 8d). We found that *Atg5* knockdown significantly upregulated the expression of oxidative stress-related protein (HO-1) and ER stress-related protein (ATF-4, Bip) compared with the APAP-only group (Fig. 8e). The western blot results of *Atg7* knockdown are similar to that of *Atg5* (Fig. S6). These results suggested that loss of autophagy gene *Atg5* or *Atg7* induces oxidative stress and ER stress, indicating a feedback mechanism of autophagy on these processes. RTCA and immunoblot analysis of Bcl-xl and cleaved CASP3 showed that, compared with the APAP-only group, apoptotic cell death was significantly increased in the *Atg5* siRNA+APAP and *Atg7* siRNA+APAP groups (Fig. 8b, c, e). These results demonstrated that autophagy plays an important role in APAP-induced apoptotic cell death in HEI-OC1 cells after APAP injury.

## Discussion

HCs in the cochlea play a critical role in converting mechanical sound waves into neural signals, and SGNs transmit these signals to the auditory cortex, resulting in hearing^15,49,50^; thus HCs and SGNs are critical for hearing ability. In the inner ear of mammals, HCs are vulnerable to multiple types of damage, and these cells lack the ability to regenerate. Therefore, dead HCs cannot be spontaneously regenerated^51–56^, which serves as the major cause of the hearing loss induced by gene mutation, noise, ototoxic drugs, inflammation, and aging^15,57–60^. In this study, we investigated the molecular mechanisms of APAP-induced ototoxicity using two in vitro models: HC-like HEI-OC1 cells and whole-organ primary culture of the murine organ of Corti.

In the inner ear, apoptosis of HCs can be activated in response to various types of damage, including acoustic trauma, ototoxicity, inflammation, and aging^61–65^. In this study, we showed that APAP induced apoptotic cell death in HEI-OC1 cells and in mouse cochlear HCs. After treatment with APAP, the HEI-OC1 cell viability was markedly decreased, but the percentage of dead cells was not very high. These results may suggest that APAP exposure decreases cell division, a phenomenon that has been previously reported^30^.

Noise, ototoxic drugs, inflammation, and aging can all increase the accumulation of mitochondrial ROS, leading to mtDNA damage, oxidative stress, and ER stress in HCs, causing HC apoptosis^16,23,61,63^. We observed that APAP induced oxidative stress, ER stress, and apoptosis in HEI-OC1 cells and cultured cochlear explants, which is consistent with previous reports^29,30^. Kalinec et al. reported that the toxic effects of APAP in HEI-OC1 cells involve the induction of ER stress signaling by controlling the PERK-mediated branch of this signaling pathway^29^. They suggested that the IRE1α-mediated ER stress pathway is not involved in APAP or NAPQI cytotoxicity. They found that HEI-OC1 cells exposed to APAP showed no clear signals of XBP-1 splicing, the downstream of IRE1α signaling. However, in this study, we showed that the IRE1α-mediated ER stress pathway is involved in APAP ototoxicity. The protein expression of XBP-1s was transiently enhanced at 6 h, and the levels of XBP-1 mRNA splicing were increased in a time-dependent manner in the early time points after APAP treatment.

APAP-induced ototoxicity has been linked to autophagy^30^. In this study, we found that treatment with APAP caused autophagy impairment as shown by the accumulation of p62. We continued to show that APAP-induced autophagy impairment was due to the reduced lysosomal activity but not formation of autophagosome. By applying LysoTracker, Magic Red, and acridine orange staining, we found that the accumulation of large liposomal vesicles increased significantly in APAP-treated cells at 24 h. Similarly, a disruption in autophagy is also associated with the accumulation of large vesicular structures in the inner segments of cone photoreceptors, increases in enlarged acidic vesicles, and abnormal late endosomes in zebrafish lacking polyphosphoinositide phosphatase synaptojanin 1^66^. Researchers have demonstrated that lysosomal dysfunction is more directly responsible for autophagy impairment^12^. We also found that the protein expression of lysosomal proteins, LAMP1 and LAMP2, was decreased after APAP treatment in both HEI-OC1 cells and cochlear HCs. We further showed that inhibition of lysosomal activity by CQ further aggravated autophagy impairment and increased apoptotic cell death. Therefore, we suggest that APAP may impair degradation of autophagosome cargo through lysosome dysfunction in HEI-OC1 cells and cochlear HCs.

In this study, we investigated three cell-signaling pathways (oxidative stress, ER stress, and autophagy) in the context of APAP-induced ototoxicity. We suggest that the three cell-signaling pathways are all involved in APAP-induced ototoxicity. It has been reported that ROS-induced mitochondrial damage may be an important upstream activator of mitophagy, a process for the selective autophagic degradation of mitochondria^67^. In this study, we found that ROS signaling is involved in APAP-induced autophagy impairment because antioxidant NAC treatment partially alleviated APAP-induced autophagy impairment as shown by the reduction of LC3-II and p62 protein levels. Autophagy impairment with lysosomal dysfunction is an important characteristic of oxidative stress-induced senescence^12^. Emerging evidence suggests that ER stress can trigger autophagy^68,69^. However, our study showed that ER stress only have a weak, if any, contribution to APAP-induced autophagy impairment, because the effect of ER inhibitor on the alleviation of autophagy impairment is not obvious.

Previous studies have shown that there is a balance between oxidative stress and autophagy in sensory HCs^13^. In this study, we showed that knockdown of *Atg5* or *Atg7* decreased the expression of LC3-II and increased APAP-induced ROS levels and apoptotic cell death. As previously reported, there is a negative feedback mechanism between ER stress and autophagy^70^. Our results showed that, when *Atg5* or *Atg7* were knocked down in HEI-OC1 cells, APAP-induced ER stress levels and apoptotic cell death were increased. These results suggested that downregulation of autophagy leads to elevated ROS and ER stress levels, which further enhances apoptosis after APAP injury.

In summary, our study demonstrates that lysosomal impairment is an important factor contributing to APAP-induced cell death in HEI-OC1 cells and mice cochlear explants with impaired autophagy (Fig. S7). These findings highlight the potential of specific lysosomal activators in alleviating APAP-induced ototoxicity. Lysosome dysfunction may serve as a candidate target for therapeutic intervention, and future studies are warranted to design and test potential therapeutic treatments for preventing hearing loss caused by APAP.

## Supplementary information

Supplementary Figure Legends

Figure S1

Figure S2

Figure S3

Figure S4

Figure S5

Figure S6

Figure S7
