# Supplementary material for: Autophagy impairment as a key feature for acetaminophen-induced ototoxicity
Source: Cell Death Dis. 2021 Jan 4;12(1):3. doi: 10.1038/s41419-020-03328-6 (PMC7791066; doi:10.1038/s41419-020-03328-6)
Supplement: Supplementary file 1 — Supplementary Figure Legends [file 41419_2020_3328_MOESM1_ESM.docx]

**Supplemental data**

**Supplemental methods**

**pTFEB-GFP** **plasmid transfection**

For monitoring lysosome biogenesis, HEI-OC1 cells were transfected with pTFEB-GFP plasmid (a gift from Dr. Andrea Ballabio, Telethon Institute of Genetics and Medicine, Italy) using Lipofectamine 3000 transfection reagent (Thermofisher, #L3000015) for 48 h. After the designated treatments, the cells were fixed with 4% paraformaldehyde fix solution and examined under a confocal microscope.

**XBP-1 mRNA splicing assay**

The XBP-1 mRNA splicing assay was done as previously described[^1^](#_ENREF_1). Briefly, total RNA was extracted from HEI-OC1 cells using TRIzol Reagent (ThermoFisher Scientific, 15596026) following the manufacturer’s protocol. RNA quality and concentration were measured by a NanoDrop 2000c spectrophotometer (ThermoFisher Scientific, Wilmington, DE, USA). The cDNA was synthesized with PrimeScript^TM^ RT reagent Kit with gDNA Eraser (TaKaRa, RR047A). Primers (5’-ACACGCTTGGGAATGGACAC-3’ and 5’-CCATGGGAAGATGTTCTGGG-3’) encompassing the spliced sequences in XBP-1 mRNA were used for the PCR amplification. The PCR was performed on a T100^TM^ Thermal Cycler (Bio-Rad, Hercules, CA, USA) using the Taq PCR Master Mix (TaKaRa, RR058A). The thermal cycling parameters were an initial denaturation step at 94°C for 2 min followed by 38 cycles of denaturation at 94°C for 20 s, annealing at 55°C for 20 s, extension at 70°C for 30 s, and a final extension at 72°C for 5 min. PCR products were separated by electrophoresis through a 2.5% agarose gel and visualized by ethidium bromide staining.

**Reference**

1 Martinon, F., Chen, X., Lee, A. H. & Glimcher, L. H. TLR activation of the transcription factor XBP1 regulates innate immune responses in macrophages. *Nat. Immunol.* **11**, 411-418, doi:10.1038/ni.1857 (2010).

**Supplementary Figure Legends**

**Fig. S1 APAP induced ER-stress in HEI-OC1 cells.** (**a**) HEI-OC1 cells were treated with 20 mM APAP for the indicated periods of time (6, 12, 24 h) or vehicle (1% DMSO) for 24 h. Cells were collected and cell lysates were subjected to western blot with anti-BiP, CHOP, XBP-1s, ATF-4 and β-actin. The right panel shows the results of densitometric analysis **P* < 0.05 vs. control cells. (**b**) RT-PCR analysis of XBP-1 mRNA splicing in HEI-OC1 cells treated with 20 mM APAP for 1, 3, 6 h, vehicle for 6 h or 5 μg/ml tunicamycin (TM, ER stress inducer) treated for 24 h as a positive control. The right panel shows the quantification of the percentage of XBP-1 mRNA splicing calculated after the densitometric analysis. **P* < 0.05 vs. control cells.

**Fig. S2 APAP induced autophagosome accumulation in HEI-OC1 cells.** (**a**) Images of GFP-LC3 in HEI-OC1 cells, n=5. (**b**) Quantification of the GFP-LC3 fluorescent puncta in (**a**). **P* < 0.05 vs. control cells.

**Fig. S3** Western blot analyses showed decreased level of the expression of SNAP29 and no alterations in Rab7, Syntaxin 17 and VAMP8 levels in HEI-OC1 cells treated with 20mM APAP for different exposure times (6, 12, 24 h) or vehicle (1% DMSO) for 24 h. β-actin served as the sample loading control.

**Fig. S4 APAP induced TFEB activation in HEI-OC1 cell.** (**a**) Western blotting was performed with TFEB, histone (nuclear marker), and tubulin (cytosol marker) antibodies. Densitometric quantification of western blot bands is shown in the right panel. **P* < 0.05 vs. control cells. Cyto, cytosolic fraction; Nu, nuclear fraction. Images shown are representative of 3 independent experiments. (**b**) HEI-OC1 cells were transfected with pTFEB-GFP plasmid and treated with or without APAP for 6 or 24h. DAPI was used to counterstain nuclei. Representative images are shown. (**c**) Fluorescence intensities were quantified from (**b**). **P* < 0.05 vs. control cells.

**Fig. S5** NAC alleviated APAP-induced ER stress as it reduced the expression of BiP, ATF-4 and CHOP.

**Fig. S6** Western blot analysis showed that Atg7 Knockdown affects APAP-induced ER-stress, oxidative stress and cell viability.

**Fig. S7 A schematic showing a proposed pathway for the APAP-induced ototoxicity though autophagy.** APAP may induce ER stress and oxidative stress, which inhibit lysosome function, leading to decreased autophagic clearance and apoptosis.

| **Gene name** | **Forward primer** | **Reverse primer** |
| --- | --- | --- |
| *LC3-GFP*(mutant)  GAPDH | 5’-CATGGACGAGCTGTACAAGT-3’  5’-AGGTCGGTGTGAACGGATTTG-3’ | 5’-CACCGTGATCAGGTACAAGGA-3’  5’-TGTAGACCATGTAGTTGAGGTCA-3’ |

**Table 1 Primers used for genotyping**
