# Supplementary figures and images for: Autophagy impairment as a key feature for acetaminophen-induced ototoxicity
Source: Cell Death Dis. 2021 Jan 4;12(1):3. doi: 10.1038/s41419-020-03328-6 (PMC7791066; doi:10.1038/s41419-020-03328-6)

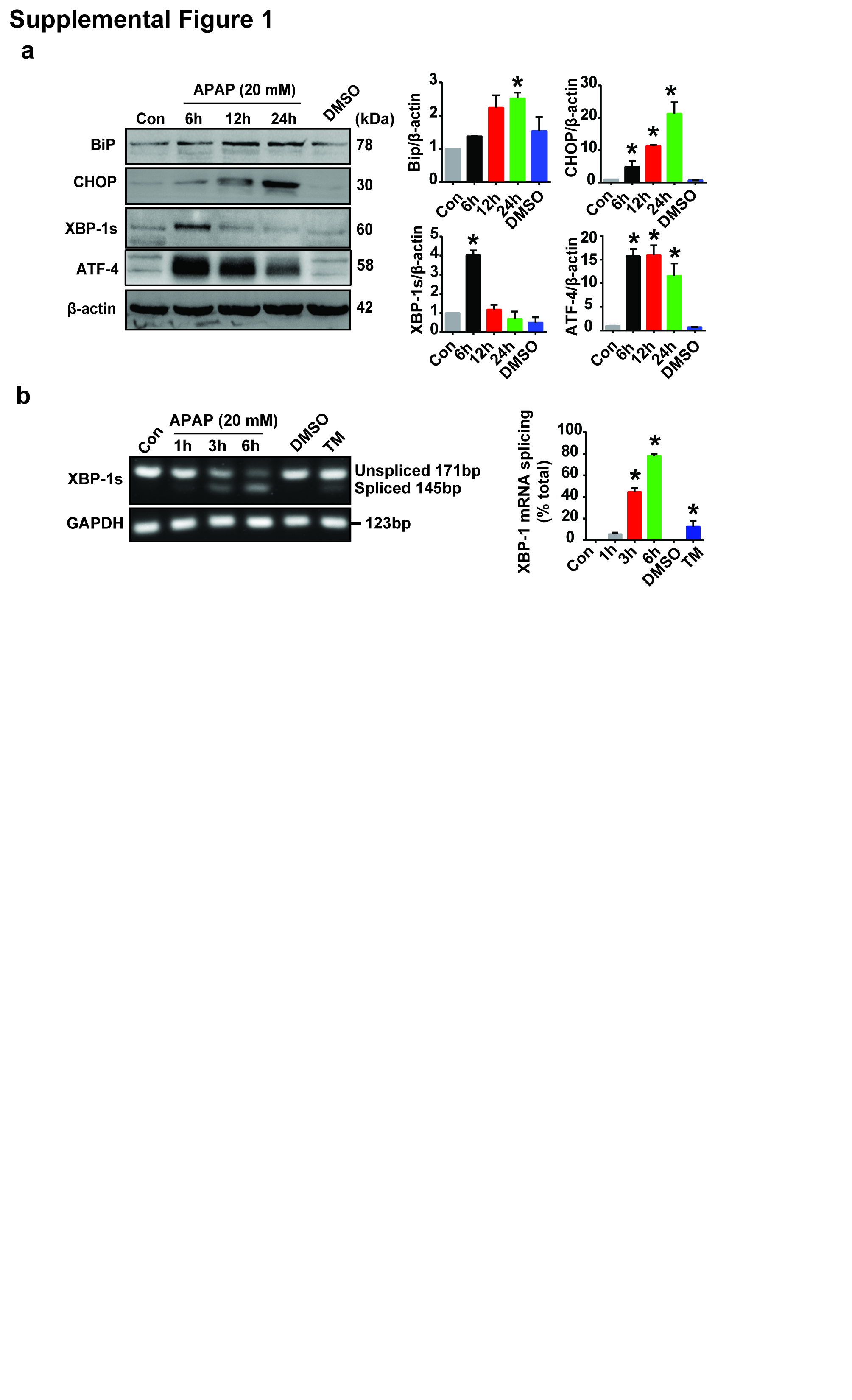

Supplement: Supplementary file 2 — Figure S1 [file 41419_2020_3328_MOESM2_ESM.tif]

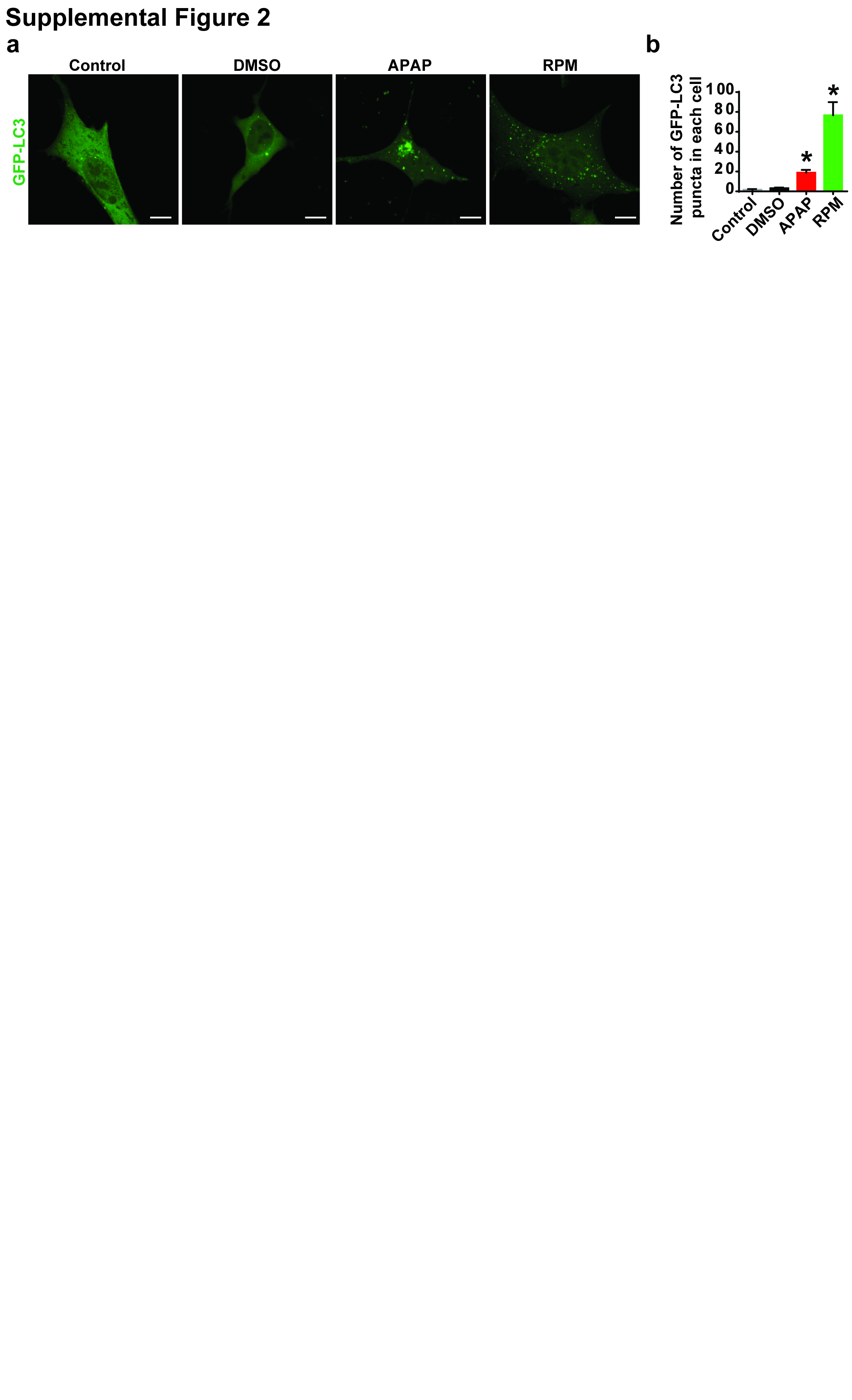

Supplement: Supplementary file 3 — Figure S2 [file 41419_2020_3328_MOESM3_ESM.tif]

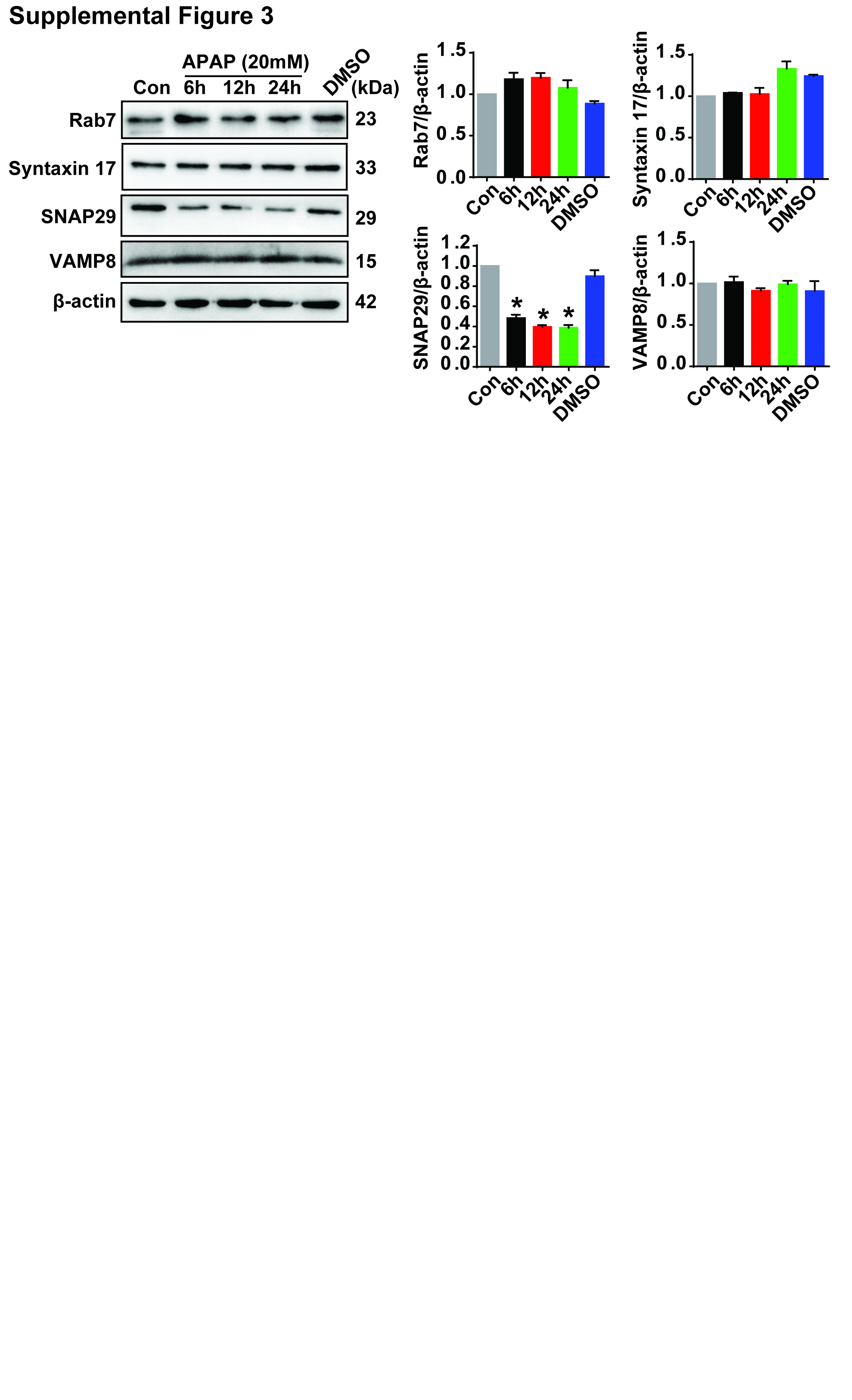

Supplement: Supplementary file 4 — Figure S3 [file 41419_2020_3328_MOESM4_ESM.tif]

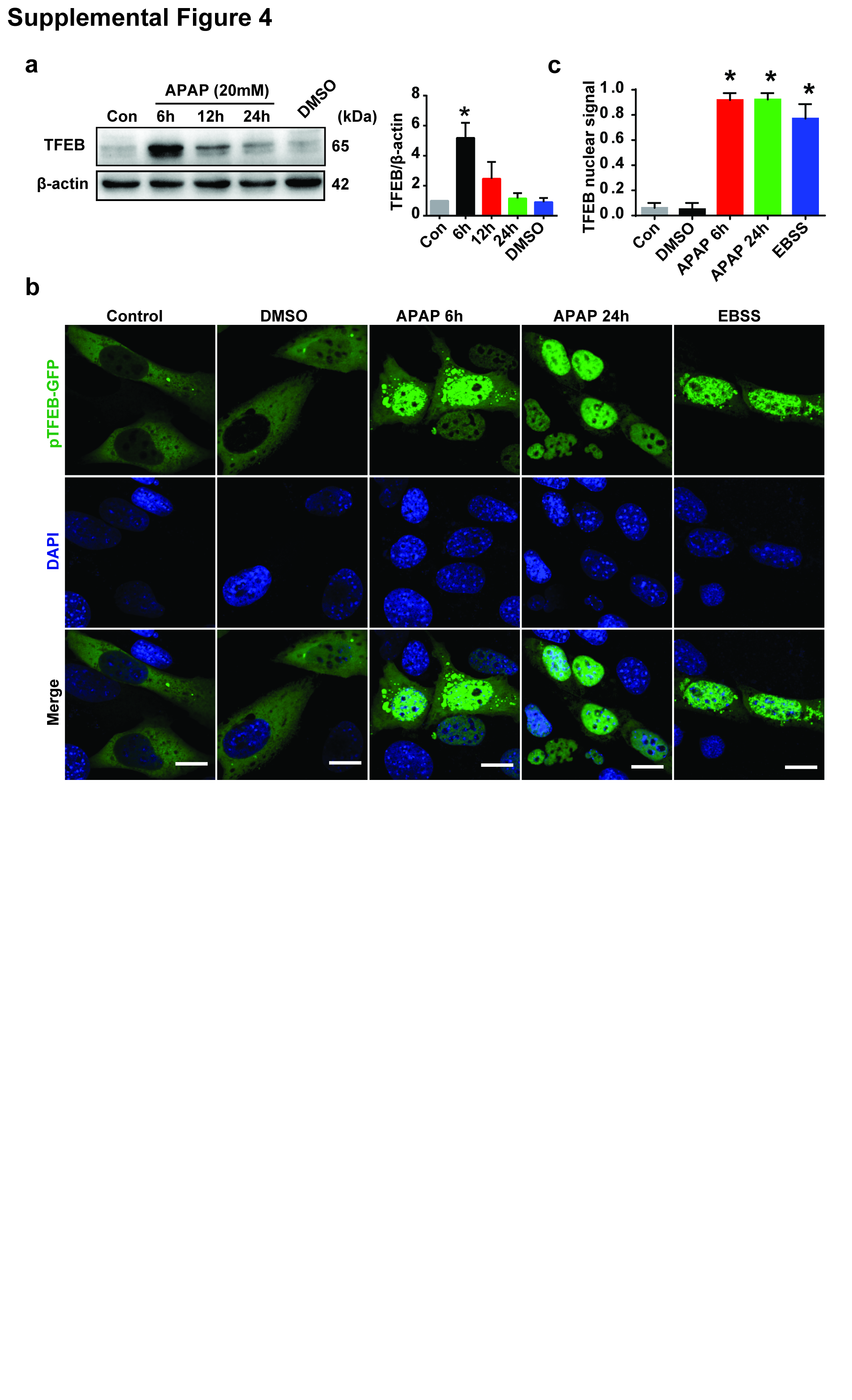

Supplement: Supplementary file 5 — Figure S4 [file 41419_2020_3328_MOESM5_ESM.tif]

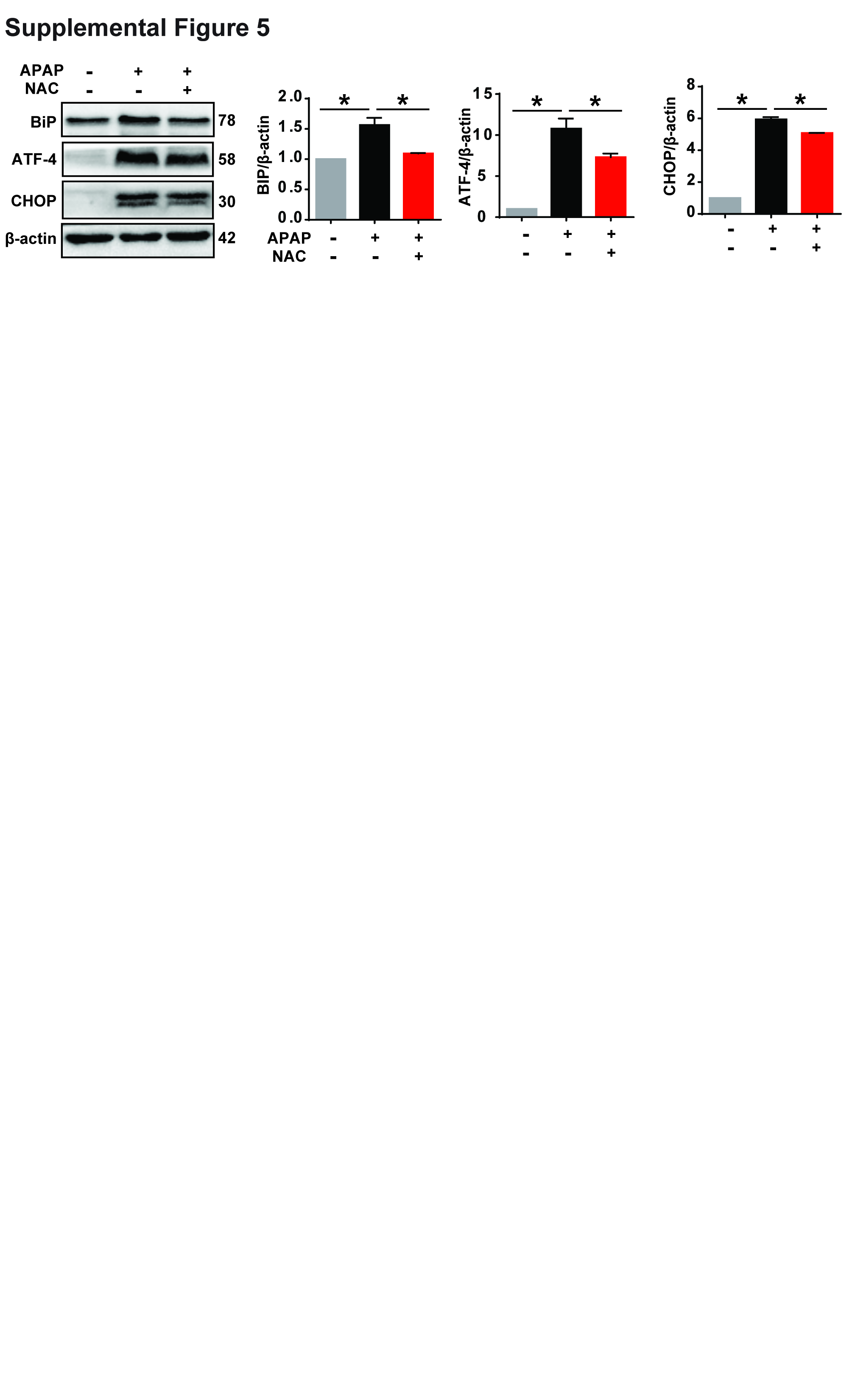

Supplement: Supplementary file 6 — Figure S5 [file 41419_2020_3328_MOESM6_ESM.tif]

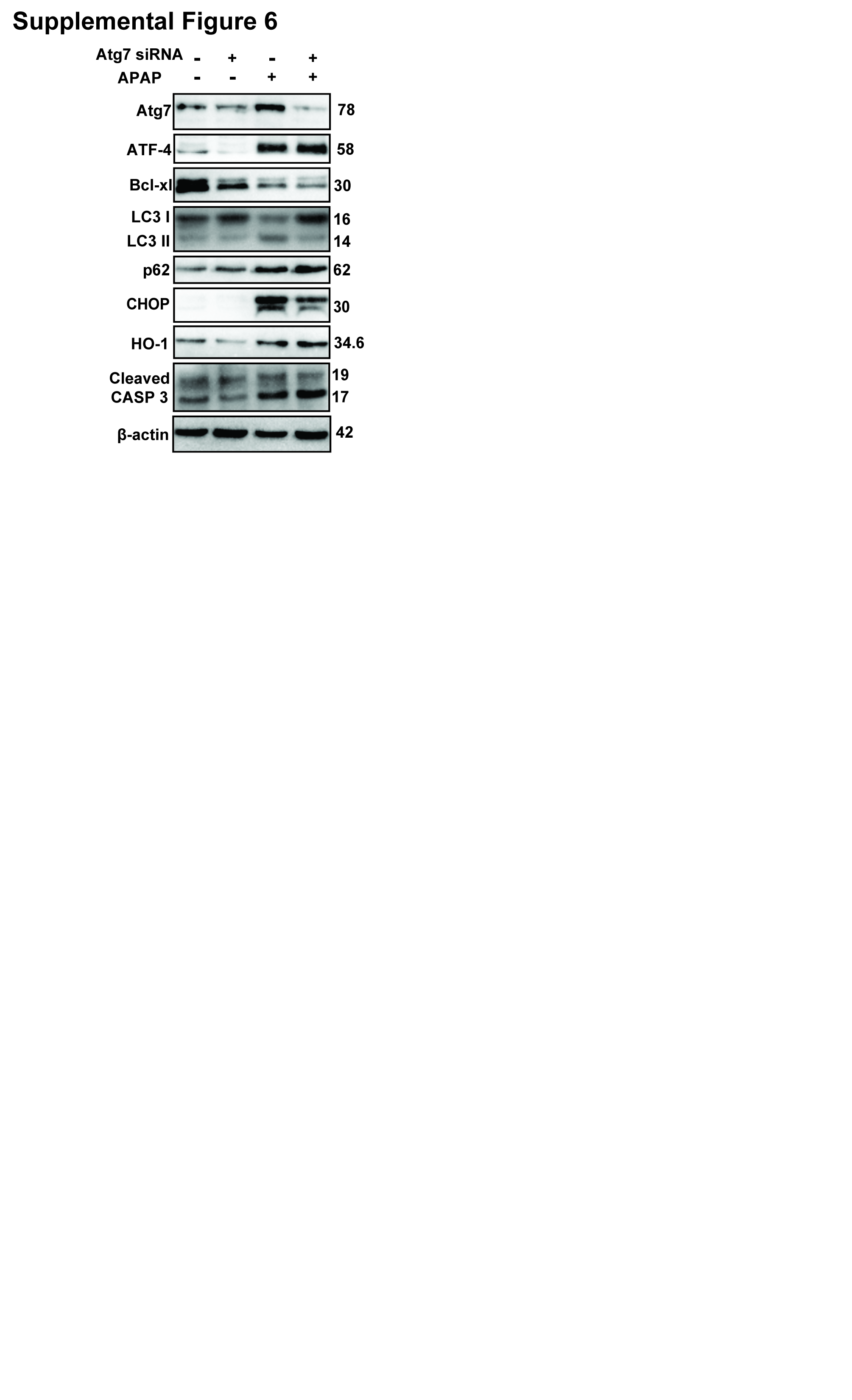

Supplement: Supplementary file 7 — Figure S6 [file 41419_2020_3328_MOESM7_ESM.tif]

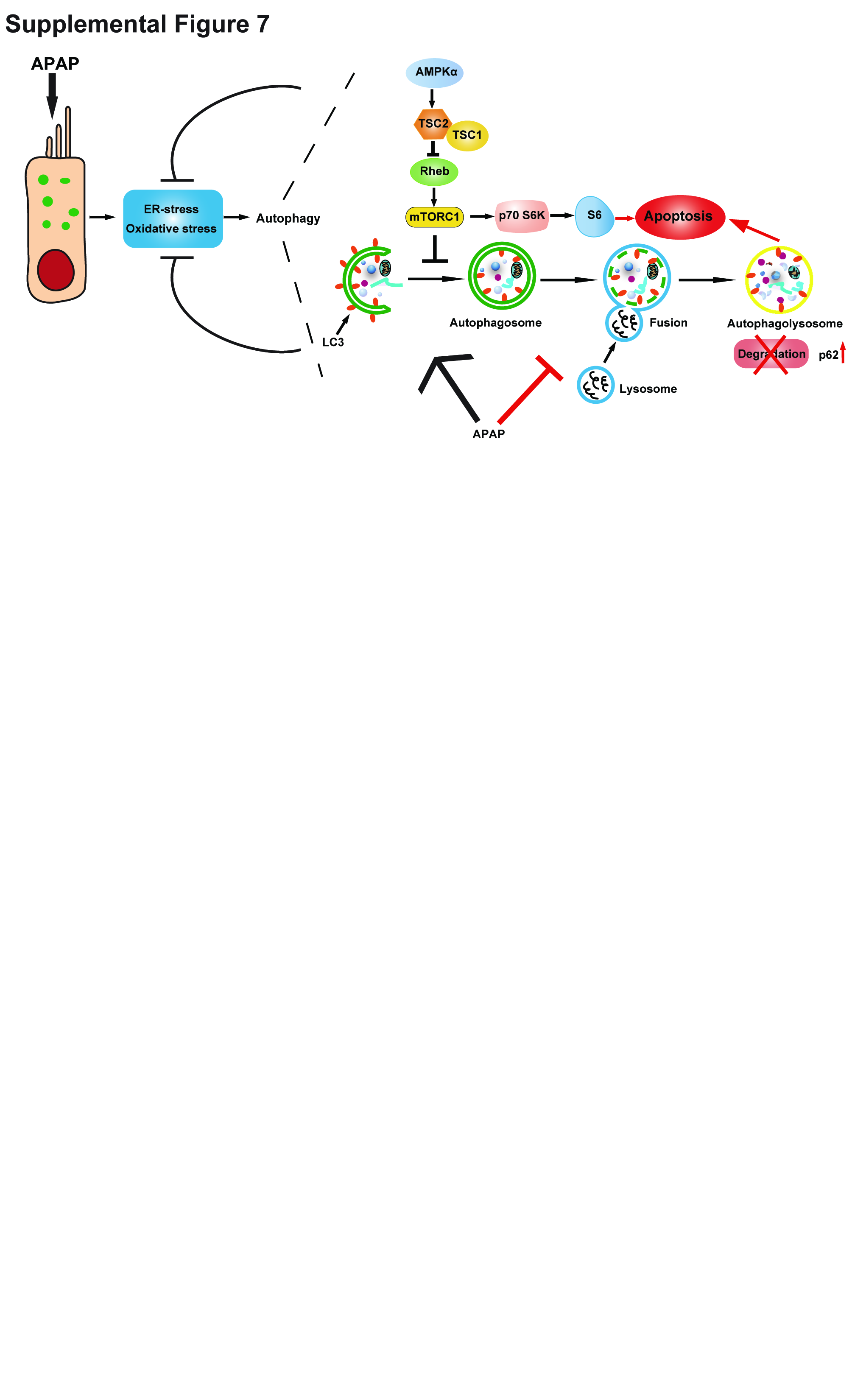

Supplement: Supplementary file 8 — Figure S7 [file 41419_2020_3328_MOESM8_ESM.tif]
